# An Aqueous Extract of *Beta vulgaris* subsp. Vulgaris Beetroot Group Reduces Lipid Accumulation in Human Keratinocyte Cells

**DOI:** 10.3390/ijms27114816

**Published:** 2026-05-27

**Authors:** Elisa Bisconti, Fabrizio Barozzi, Erika Stefàno, Ilaria Serra, Francesco Vari, Giulia Vergine, Marina Damato, Rocco Placì, Francesco Paolo Fanizzi, Dario Domenico Lofrumento, Gian Pietro Di Sansebastiano, Francesca Baldassarre, Daniele Vergara, Anna Maria Giudetti, Giuseppe Ciccarella

**Affiliations:** 1 Department of Biological and Environmental Sciences and Technologies (DiSTeBA), University of Salento, 73100 Lecce, Italy; elisa.bisconti@unisalento.it (E.B.); fabrizio.barozzi@unisalento.it (F.B.); erika.stefano@unisalento.it (E.S.); ilaria.serra@uniroma1.it (I.S.); francesco.vari@unisalento.it (F.V.); giulia.vergine@unisalento.it (G.V.); rocco.placi@unisalento.it (R.P.); fp.fanizzi@unisalento.it (F.P.F.); dario.lofrumento@unisalento.it (D.D.L.); gp.disansebastiano@unisalento.it (G.P.D.S.); francesca.baldassarre@unisalento.it (F.B.); daniele.vergara@unisalento.it (D.V.); giuseppe.ciccarella@unisalento.it (G.C.); 2 Department of Physiology and Pharmacology “V. Erspamer”, Sapienza University of Rome, Ple Aldo Moro 5, 00185 Rome, Italy; 3 Department of Experimental Medicine (DiMeS), University of Salento, 73100 Lecce, Italy; marina.damato@unisalento.it; 4 Institute of Nanotechnology, National Research Council, CNR NANOTEC, Via Monteroni, 73100 Lecce, Italy

**Keywords:** keratinocytes, *Beta vulgaris*, lipid droplets, oxidative stress, lipotoxicity

## Abstract

Epidermal lipid homeostasis is crucial for skin barrier integrity. This study investigated the effects of an aqueous extract from *Beta vulgaris* subsp. *vulgaris* Beetroot Group (BvE) on stress responses and lipid metabolism in HaCaT keratinocytes. BvE, obtained from leaves grown in SETIS^®^ bioreactors as a standardized biomass source, was chemically characterized by ^1^H NMR and ^13^C NMR. HaCaT cells were treated with BvE (1 µg/mL), H_2_O_2_, or palmitic/oleic acids (PA/OA) to evaluate its protective effects against oxidative damage and lipotoxic stress. Under these conditions, BvE exhibited a distinctive dual action as a reactive oxygen species (ROS) scavenger and triacylglycerol (TAG)-lowering agent. On the one hand, BvE was associated with decreased intracellular ROS levels and changes in NRF2 protein expression, suggesting involvement of redox-regulatory pathways. On the other hand, it was associated with attenuation of lipotoxicity, as evidenced by reduced lipid droplet (LD) formation and decreased expression of DGAT1 and PLIN2. Furthermore, these effects were accompanied by a reduction in Unfolded Protein Response (UPR) markers, modulation of AMPK-associated signaling, attenuation of mitochondrial disfunction, and decreased p53 phosphorylation, findings collectively consistent with a coordinated cytoprotective response. In conclusion, BvE shows potential to protect keratinocytes against lipotoxicity and oxidative stress through mechanisms that may involve both chemical and biological antioxidant activity and metabolic reprogramming, supporting its further investigation for dermatological applications.

## 1. Introduction

The epidermis constitutes the outermost protective barrier of the human body, and its integrity depends critically on tightly regulated lipid homeostasis. The lipidome of keratinocytes, the dominant cell type of the basal layer, is composed primarily of phospholipids, cholesterol (CHOL), and triacylglycerols (TAG). During terminal differentiation into corneocytes, enzymatic cleavage of precursor lipids generates ceramides, free fatty acids (FFAs), and CHOL, which together form the extracellular lipid matrix of the stratum corneum, essential for barrier integrity [1]. Disruption of this balance carries well-documented pathological consequences, including permeability barrier dysfunction and ichthyosiform phenotypes [2]. Intracellular neutral lipids are stored in lipid droplets (LDs), dynamic organelles that participate in lipid trafficking, membrane remodelling, and cellular signalling. LD biogenesis is closely linked to neutral lipid synthesis, in which diacylglycerol acyltransferases (DGAT1 and DGAT2) play a key role by catalysing the final step of TAG synthesis [3,4].

In keratinocytes, LDs serve as a regulated reservoir of lipid precursors for barrier synthesis, and their dynamics are tightly controlled during epidermal differentiation [4]. A central regulator of this process is Perilipin 2 (PLIN2), a structural protein of the LD surface and a universally recognised marker of intracellular lipid accumulation [5]. By coating the LD phospholipid monolayer, PLIN2 physically impedes cytosolic lipases, stabilising stored TAG and reducing lipolytic flux [6]. Its expression is upregulated in keratinocytes by diverse irritants via calcium-modulated lipid redistribution [7]. AMPK-mediated phosphorylation of PLIN2, targets it for chaperone-mediated autophagic degradation in hepatocytes, adipocytes, or muscle cells [8,9]. However, PLIN2 reduction may also reflect a direct structural loss of LD stabilization and coat integrity [10]. Whether this regulatory axis operates analogously in keratinocytes, and whether it can be pharmacologically engaged in an epidermal context, remains poorly defined.

Intracellular lipid overload can induce lipotoxicity characterised by oxidative and endoplasmic reticulum (ER) stress, impaired mitochondrial function, and activation of pro-inflammatory signalling [9]. In keratinocytes, lipid accumulation couples with NLRP3 inflammasome activation, driving release of IL-1β and IL-18 [11], establishing excess neutral lipid accumulation as an active driver of epidermal stress and inflammation rather than a mere metabolic marker [12]. The clinical relevance of these mechanisms is further underscored by the association between obesity, dyslipidemia, and impaired epidermal barrier competence [13,14], conditions in which elevated circulating palmitic acid (PA) and leptin synergistically enhance keratinocyte inflammatory responses [15].

*Beta vulgaris* (red beetroot) is a rich source of betalains, polyphenols, flavonoids, ascorbic acid, and inorganic nitrate, with well-documented antioxidant, hypolipidaemic, and anti-inflammatory activities [16,17]. While in vitro-produced biomass of *B. vulgaris* has previously been established as a sustainable and standardized source of material, its use has thus far been restricted to root-derived fractions [10].

The present study proposes *B. vulgaris* leaves as a more abundant and equally sustainable alternative, capable of yielding a high-quality and compositionally stable extract, with potential applications in plant-based cosmeceutical formulations aimed at restoring epidermal lipid homeostasis. Among its bioactive constituents, betaine (N,N,N-trimethylglycine) activates hepatic AMPK, inhibiting ACC and suppressing SREBP-1c and fatty acid synthase expression, thereby reducing intracellular lipid accumulation [18]. Moreover, we also investigated the effect of an aqueous leaf extract of *Beta vulgaris* subsp. *vulgaris* Beetroot Group on antioxidant defence, intracellular LD accumulation and PLIN2 expression in HaCaT keratinocytes, hypothesising antioxidant and anti-lipogenic effects.

## 2. Results

### 2.1. Chemical Characterization of the Beta vulgaris Extract

#### 2.1.1. Betanin and Antioxidant Activity Quantification

**Figure 1:**
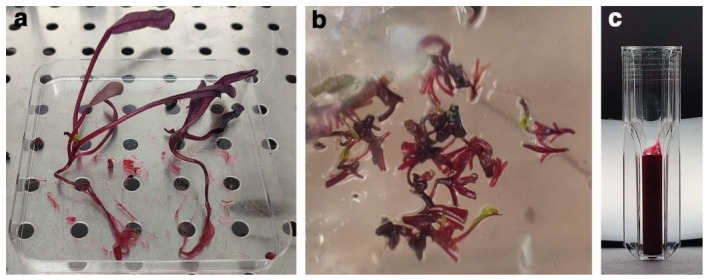
*Beta vulgaris* plants and *Beta vulgaris* extract. (**a**) *B. vulgaris* plants after 30 days of growth in SETIS^®^ bioreactor; (**b**) Sectioned apices reinserted into the SETIS^®^ bioreactor for new 30-day growth cycle; (**c**) *Beta vulgaris* Extract (BvE) obtained from lyophilized leaves.

*B. vulgaris* plants (Figure 1a) were cultivated in a SETIS^®^ bioreactor to ensure reproducible biomass production, independent of open-field cultivation or variability in commercially available plant material. After 30 days, leaves were harvested and subdivided into meristematic apices and leaf laminas. The apices were used to initiate a new growth cycle (Figure 1b), whereas the leaves were weighed (fresh weight, FW), lyophilized, weighed again (dry weight, DW) and stored at −80 °C. The plants grown in the bioreactor were characterized by a very uniform pigmentation, which was also evident at the root level. No signs of hyperhydricity were observed. The extract obtained from the leaves appeared as an intense purple-red solution (Figure 1c), visually comparable to beetroot juice.

The *Beta vulgaris* Extract (BvE) was analyzed for betanin, soluble phenols, flavonoids, nitrate content, and antioxidant activity, and the results are reported in Table 1.

The extract exhibited a betanin concentration of 0.24 ± 0.03 mg mL^−1^, corresponding to 0.52 ± 0.09 mg g^−1^ FW, which is lower than that in beetroot juice [19,20,21,22]. BvE soluble phenolics content had a value of 0.69 ± 0.12 mg Gallic Acid Equivalent (GAE) mL^−1^, equivalent to 1.38 ± 0.24 mg GAE g^−1^ FW. This value is comparable with the values obtained in beetroot juice [20,22,23,24]. Regarding the flavonoid concentration, the value obtained in BvE is 0.16 ± 0.04 mg Catechin Equivalent (CE) mL^−1^, equivalent to 0.33 ± 0.08 mg CE g^−1^ FW, which aligns with the range of values reported for beetroot juice [24]. The nitrate content of the BvE is 0.22 ± 0.08 mg mL^−1^, equivalent to 0.43 ± 0.16 mg g^−1^ FW; this is higher with respect to the range reported in previous studies [24,25] but is considered low and safe from a health perspective [26,27]. The antioxidant capacity was measured at 4.75 ± 1.14 µmol Trolox Equivalents (TE) mL^−1^, which corresponds to 10.08 ± 2.50 µmol TE g^−1^ FW. The observed antioxidant activity remains within the typical range reported for beetroot juice [19,20,21,22].

#### 2.1.2. ^1^H NMR Spectroscopy Composition

**Figure 2:**
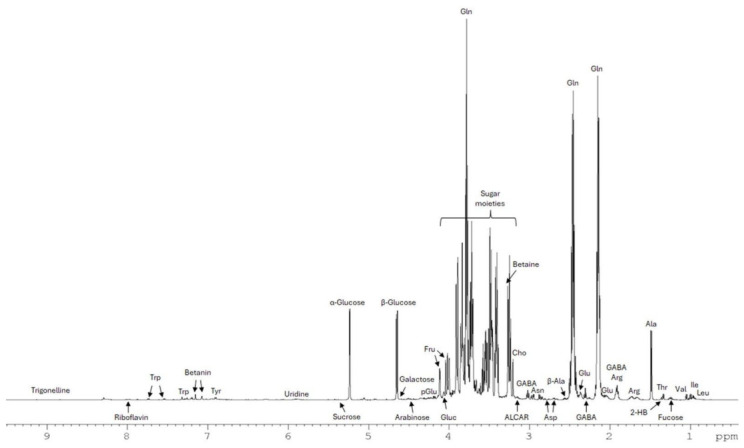
^1^H NMR spectra of *B. vulgaris* Extract (BvE) at δ 0.5–9.5 ppm (D2O). Peaks assigned in the spectrum are labelled as listed in Table S1.

The metabolites identified in the BvE by ^1^H NMR spectroscopy provide a comprehensive characterization of its chemical composition (Figure 2; Table S1). A detailed analysis of the metabolite profile of BvE was performed using 1D and 2D NMR spectra. A representative 1D ^1^H NMR spectrum (600 MHz) of BvE is reported in Figure 2. The high-field region from 0.8 to 3.2 ppm includes signals mainly consisting of amino acids, while the mid- to low-field region from 3.2 to 5.5 ppm shows peaks mainly from carbohydrates. The low-field region beyond 6.0 ppm mainly contains aromatic resonances (Figure 2; Table S1). Quantification of identified metabolites in BvE was performed using the ratio method, as signal intensity is proportional to the molar concentration of metabolites in the ^1^H NMR spectrum [28,29]. Out of the 38 identified metabolites, 31 were easily quantified, since they showed no signal overlap. Metabolite measurements were calculated as mg/mL of extract based on the integration of their corresponding peaks in the ^1^H NMR spectra, using TSP as an internal standard (Table S1). 

Amino acids detected in BvE include leucine, isoleucine, valine, threonine, alanine, arginine, glutamine, glutamate, GABA, aspartate, asparagine, and serine in the low–mid-field region, and tyrosine, phenylalanine, and tryptophan in the higher-field region. The presence of choline was suggested by its characteristic methyl peak at 3.22 ppm. The NMR analysis also suggested the presence of organic acids, including propionate, fumarate, and formate. Carbohydrates were the most abundant BvE components, with α- and β-glucose and fructose present at the highest concentrations, indicating that sugars represent a major fraction of the extract, together with betaine (Figure 2). Consistent with the known composition of beet-derived extracts, betanin was among the most abundant secondary metabolites, as indicated by the characteristic broad singlets in the ^1^H NMR spectrum at 7.15 and 7.07 ppm and the corresponding ^13^C signals at 102.5 and 116.8 ppm, respectively [30] (Table S1). In addition, trigonelline, a product from vitamin B3 metabolism, and riboflavin signals are visible in the ^1^H NMR spectrum (Figure 2; Table S1). The presence of all the metabolites suggested by the 1D ^1^H NMR spectra were further confirmed by 2D experiments: COSY, HSQC, HMBC, and J-resolved (Figures S1–S4).

### 2.2. Effects of BvE on Cell Viability 

**Figure 3:**
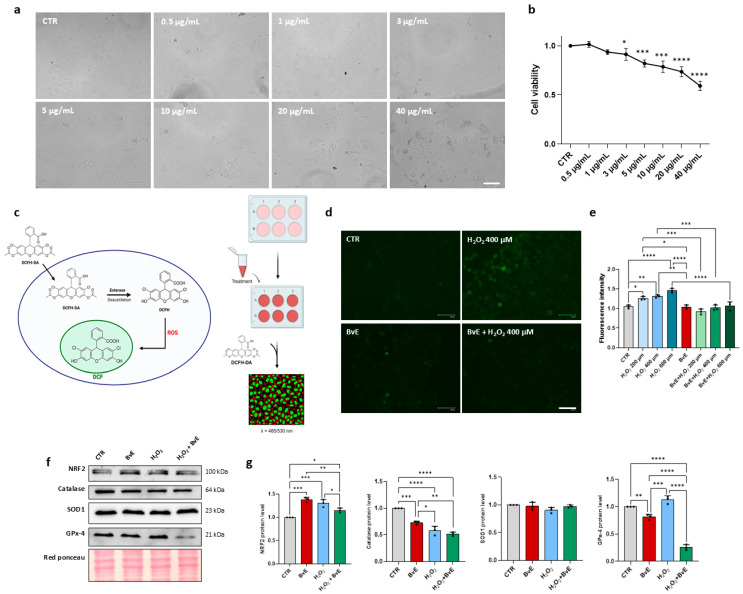
Effect of BvE on cell viability and H_2_O_2_-induced cytotoxicity in HaCaT keratinocytes. (**a**) Representative bright-field microscopy images of HaCaT cells without treatment (CTR) and after 24 h of treatment with different concentrations of *Beta vulgaris* extract (BvE) (0.5, 1, 3, 5, 10, 20, and 40 μg/mL). Scale bars 100 μm. (**b**) Cell viability assay performed on HaCaT cells treated for 24 h with BvE at concentrations of 0.5, 1, 3, 5, 10, 20, and 40 μg/mL. (**c**) Schematic representation of the DCFH-DA assay (Created in BioRender. Vergara, D. (2025) https://BioRender.com/uwqc3ic accessed on 23 May 2026). (**d**) Representative fluorescence images showing ROS level (green; stained with DCFH-DA probe) and (**e**) relative fluorescence intensity in HaCaT cell line exposed to hydrogen peroxide (H_2_O_2_; 400 µM) for 24 h in the presence or absence of *Beta vulgaris* extract (BvE; 1 µg/mL). Fluorescence intensity was normalized to protein content. Scale bars 100 μm. (**f**) Western blot analysis of NRF2, Catalase, SOD1, and GPx-4 protein levels and (**g**) relative quantification in HaCaT cells treated with BvE (1 µg/mL), H_2_O_2_ (400 µM), and co-treatment of BvE + H_2_O_2_. Ponceau S staining was used as a total protein loading control. Data are shown as mean ± SD of at least three independent experiments (* *p* < 0.05; ** *p* < 0.01; *** *p* < 0.005; **** *p* < 0.001).

To establish a safe and biologically relevant concentration range for subsequent assays, we first evaluated the cytotoxic profile of BvE on human epidermal keratinocytes (HaCaT cells). Cells were exposed to increasing concentrations of BvE (0.5–40 µg/mL) for 24 h. Phase-contrast microscopy revealed progressive morphological alterations at higher concentrations, including cell rounding and detachment, indicative of cytotoxic stress (Figure 3a). In line with these observations, cell viability assessment showed a dose-dependent reduction, with statistically significant decreases emerging from 3 µg/mL and reaching approximately 60% viability at the highest tested concentration of 40 µg/mL (Figure 3b). To determine whether this cytotoxic profile was conserved across different epithelial contexts, the same experimental conditions were applied to HCT15 cells, a human colorectal epithelial cell line. HCT15 cells exhibited an analogous dose-dependent reduction in viability, with a cytotoxic profile comparable to that observed in HaCaT cells (Figure S5a,b). Based on these findings, a concentration of 1 µg/mL BvE, which preserved full cell viability in both cell models, was selected for all subsequent experiments.

### 2.3. BvE Reduces Oxidative Stress in Keratinocytes

Given the significant antioxidant capacity of BvE, we next investigated whether this property translated into biologically relevant cytoprotective effects at the cellular level. HaCaT keratinocytes were challenged with increasing concentrations of hydrogen peroxide (H_2_O_2_; 200, 400, and 600 µM), a well-established inducer of intracellular oxidative stress widely used in in vitro stress models, in the presence or absence of BvE (1 µg/mL) for 24 h. Phase-contrast microscopy revealed that H_2_O_2_ alone induced progressive, dose-dependent morphological alterations, including cell rounding and detachment, which were markedly attenuated by BvE co-treatment at 200 and 400 µM H_2_O_2_ (Figure S6a,b). Cell viability analysis corroborated these observations, showing that H_2_O_2_ significantly reduced viability, compared with the control (CTR) at all tested concentrations (200 µM, ** *p* < 0.01; 400 µM, ** *p* < 0.01; 600 µM, **** *p* < 0.001), and that co-treatment with BvE significantly restored viability relative to the respective H_2_O_2_ condition (Figure S6a,b).

To identify intracellular oxidative burden, we employed the cell-permeable fluorescent probe DCFH-DA. Upon passive diffusion into the cell, DCFH-DA is deacetylated by cytoplasmic esterases to yield the non-fluorescent intermediate DCFH, which is subsequently oxidized by intracellular ROS to the highly fluorescent compound DCF (2′,7′-dichlorofluorescein), providing a real-time readout of the intracellular redox environment (Figure 3c). Fluorescence microscopy revealed a marked increase in DCF signal in H_2_O_2_-treated cells relative to untreated controls, whereas co-treatment with BvE consistently reduced fluorescence to levels comparable to CTR (Figure 3d). Quantitative analysis confirmed a significant, dose-dependent elevation in intracellular ROS across all H_2_O_2_ concentrations tested. Notably, BvE co-treatment significantly attenuated H_2_O_2_-induced oxidative stress at all concentrations (* *p* < 0.05 to **** *p* < 0.001), restoring DCF fluorescence intensity to near-basal levels (Figure 3e).

### 2.4. Molecular Mechanism of the Antioxidant Effect of BvE

To elucidate the molecular mechanisms underlying the antioxidant effects of BvE, HaCaT keratinocytes were exposed to BvE (1 μg/mL), H_2_O_2_ (400 μM for 24 h), or their combination. The expression levels of key redox-regulatory proteins were subsequently evaluated by Western blot analysis (Figure 3f,g).

As shown in Figure 3f,g, exposure to H_2_O_2_ significantly increased NRF2 protein levels compared to the CTR group (**** *p* < 0.001), indicating activation of the oxidative stress-responsive antioxidant pathway. Treatment with BvE alone also induced a statistically significant upregulation of NRF2 relative to CTR (* *p* < 0.05), suggesting an intrinsic antioxidant or cytoprotective potential of the extract. Notably, co-treatment with H_2_O_2_ and BvE resulted in NRF2 levels that remained significantly elevated compared to CTR (* *p* < 0.05) but were moderately reduced relative to H_2_O_2_ treatment alone. This pattern may indicate a modulatory effect of BvE on NRF2 activation under oxidative stress conditions, potentially preventing excessive activation.

Catalase protein expression was significantly decreased following treatment with BvE (*** *p* < 0.005), H_2_O_2_ (**** *p* < 0.001), and their combination (**** *p* < 0.001 vs. CTR), with no statistically significant differences observed between the H_2_O_2_ and H_2_O_2_ + BvE groups. In contrast, SOD1 protein levels remained unchanged across all experimental conditions, indicating that this enzyme is not significantly affected by either oxidative stress or BvE treatment in this model.

Regarding GPx-4, BvE alone induced a significant downregulation compared to CTR (** *p* < 0.01), whereas H_2_O_2_ treatment resulted in a marked increase in GPx-4 expression (*** *p* < 0.005 vs. BvE). Importantly, co-treatment with H_2_O_2_ and BvE led to a pronounced reduction in GPx-4 levels compared to all other conditions (**** *p* < 0.001 vs. H_2_O_2_, BvE, and CTR), suggesting a strong regulatory effect of BvE on this enzyme under oxidative stress.

Collectively, these findings indicate that BvE exerts a significant antioxidant effect in HaCaT keratinocytes exposed to H_2_O_2_-induced oxidative stress, primarily through modulation of the NRF2 signaling pathway and selective regulation of GPx-4 expression. 

### 2.5. BvE Counteracts Pathological Lipid Accumulation and Attenuates ER Stress in Epithelial Cells

Keratinocytes rely on tightly regulated lipid metabolism for epidermal barrier integrity, and excess saturated fatty acids are known to trigger lipotoxic cascades, including oxidative stress and ER stress via Unfolded Protein Response (UPR) activation, that underline several inflammatory skin conditions [12]. Given that BvE demonstrated the ability to modulate oxidative damage in keratinocytes, we next investigated whether it could interfere with both intracellular lipid accumulation and the downstream ER stress response.

**Figure 4:**
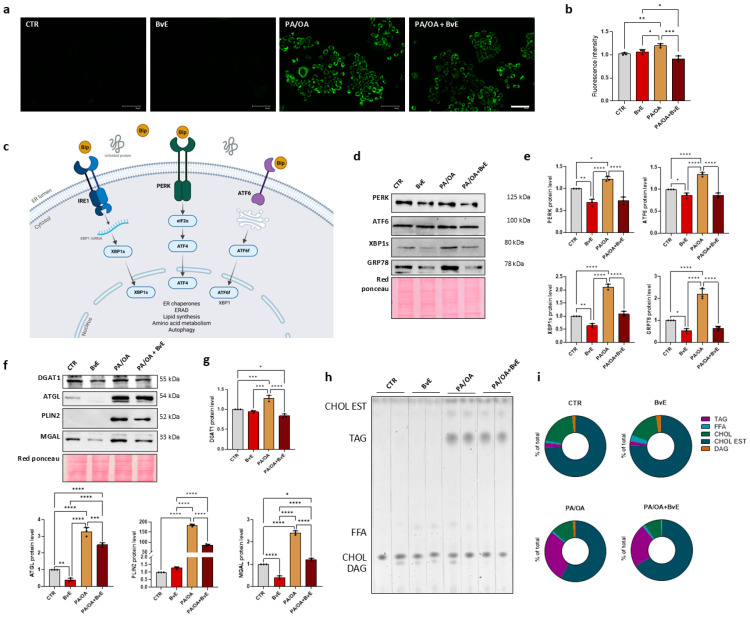
BvE modulates ER Stress and lipid metabolism in HaCaT Cells. (**a**) Representative fluorescence images showing lipid droplets (LDs, green; stained with BODIPY probe) and (**b**) relative fluorescence intensity in HaCaT cell line treated with *Beta vulgaris* extract (BvE; 1 μg/mL) for 24 h, palmitic acid (PA) and oleic acid (OA) for 48 h (PA/OA; 1:1 ratio, 200 µM each), and pre-treatment of PA/OA for 48 h + BvE for 24 h (co-treatment PA/OA + BvE). Fluorescence intensity was normalized to protein content. Scale bars 100 μm. (**c**) Schematic representation of Unfolded Protein Response (UPR). /Created in BioRender. Vergara, D. (2025) https://BioRender.com/uwqc3ic). (**d**) Western blot analysis of PERK, ATF6, XBP1s, and GRP78 protein levels and (**e**) relative quantification in HaCaT cells treated with BvE (1 μg/mL), PA/OA (1:1 ratio, 200 µM each), and PA/OA + BvE. Ponceau S staining was used as a total protein loading control. (**f**) Western blot analysis of DGAT1, ATGL, PLIN2, and MAGL protein levels and (**g**) relative quantification in HaCaT cells treated with BvE (1 μg/mL), PA/OA (1:1 ratio, 200 µM each), and PA/OA + BvE. Ponceau S staining was used as a total protein loading control. (**h**) Thin-layer chromatography (TLC) analysis and (**i**) relative quantification of intracellular lipid classes, including CHOL EST (cholesterol ester), TAG (triacylglycerol), FFA (free fatty acid), CHOL (cholesterol), and DAG (diacylglycerol) in the HaCaT cell line. Each lipid species is expressed as a percentage of the total neutral lipid content. Data are shown as mean ± SD of at least three independent experiments (* *p* < 0.05; ** *p* < 0.01; *** *p* < 0.005; **** *p* < 0.001).

To induce a lipotoxic state, HaCaT cells were challenged with a combination of PA and oleic acid (OA), two fatty acids widely used to recapitulate pathological intracellular LD accumulation in vitro [31]. Cells were treated with PA/OA for 48 h in the presence or absence of BvE (1 µg/mL), added during the last 24 h of incubation. Intracellular lipid content was assessed using the BODIPY fluorescent probe by fluorescence microscopy (Figure 4a,b). PA/OA treatment induced a striking accumulation of intracellular LDs compared to control cells, while cells co-treated with BvE displayed fluorescence levels comparable to untreated controls, suggesting a marked anti-adipogenic effect of the extract. Measurement of fluorescence confirmed that PA/OA significantly increased BODIPY staining compared to both CTR (** *p* < 0.01) and BvE-treated cells (* *p* < 0.05), while PA/OA + BvE co-treatment significantly reduced lipid accumulation compared to PA/OA alone (*** *p* < 0.005), restoring fluorescence to levels at or below those of untreated cells (Figure 4b). It should be noted that BvE likewise reduced PA/OA-induced lipid accumulation in another epithelial cell model, HCT15 intestinal cells (Figure S7a,b), indicating that this property is not restricted to keratinocytes.

We next examined whether BvE could also regulate the ER stress response elicited by lipid overload. The UPR operates through three main signaling branches, IRE1α/XBP1s, PERK/eIF2α/ATF4, and ATF6, schematically illustrated in Figure 4c. PA/OA treatment significantly increased the protein levels of PERK, ATF6, XBP1s, and the ER chaperone GRP78 compared to untreated controls, confirming that lipid overload is sufficient to activate all three UPR branches in keratinocytes (Figure 4d,e). Notably, BvE alone (1 µg/mL) significantly reduced the basal expression of all four UPR markers, while co-incubation with PA/OA effectively reduced lipotoxicity-induced ER stress, restoring PERK (**** *p* < 0.001), ATF6 (**** *p* < 0.001), XBP1s (**** *p* < 0.001), and GRP78 (**** *p* < 0.001) protein levels compared with PA/OA alone. A consistent pattern of UPR downregulation upon BvE treatment was also observed in HCT15 intestinal epithelial cells (Figure S5c,d), suggesting that the capacity of BvE to attenuate ER stress represents a broader property of the extract across epithelial tissues.

### 2.6. Mechanism of BvE-Regulated LD Dynamics in Keratinocytes

To elucidate the mechanisms underlying the anti-adipogenic effect of BvE, the expression of key LD remodelling enzymes was assessed by Western blot (Figure 4f,g).

LD turnover is tightly regulated by a coordinated series of enzymatic activities. LD formation is primarily driven by enzymes involved in TAG synthesis, including DGAT1 and DGAT2, while LD degradation proceeds through a sequential lipolytic cascade mediated by adipose triglyceride lipase (ATGL/PNPLA2) and monoacylglycerol lipase (MAGL), which complete fatty acid liberation by releasing FFA and glycerol. The structural integrity and growth of LDs are further governed by PLIN2, an LD-coating protein that controls lipase accessibility to the lipid core.

Analysis of LD-associated proteins revealed a comprehensive modulation of lipid metabolism by BvE. DGAT1 was significantly upregulated by PA/OA treatment compared to control (*** *p* < 0.005), but markedly reduced upon BvE co-treatment relative to both control (* *p* < 0.05) and PA/OA alone (**** *p* < 0.001) (Figure 4f,g), suggesting that BvE limits LD biogenesis at the level of lipid esterification. PLIN2 was dramatically upregulated by PA/OA (**** *p* < 0.001) and significantly reduced by BvE co-treatment (**** *p* < 0.001 vs. PA/OA), indicating a decreased stabilization and coating of newly formed LDs.

BvE also modulated the expression of lipolytic enzymes. ATGL levels were modestly reduced compared with the control (** *p* < 0.01) by BvE alone and significantly increased by PA/OA treatment (**** *p* < 0.001); however, BvE co-treatment markedly attenuated this increase compared with PA/OA alone (*** *p <* 0.005) (Figure 4f,g). Similarly, MAGL expression was significantly reduced by BvE alone compared to control (**** *p* < 0.001), markedly induced by PA/OA (**** *p* < 0.001), and substantially restored toward basal levels by PA/OA + BvE co-treatment (**** *p* < 0.001 vs. PA/OA).

### 2.7. BvE Induces Lipid Class Redistribution in HaCaT Keratinocytes

To characterize the changes in intracellular lipid composition induced by BvE, a thin-layer chromatography (TLC)-based lipid profiling approach was employed. TLC separation of total lipid extracts revealed distinct differences in the lipid class distribution across experimental conditions (Figure 4h,i). Semiquantitative analysis of the relative lipid class distribution, expressed as a percentage of total lipid content, confirmed these observations (Figure 4i). In CTR cells, the lipid profile was dominated by CHOL esters (74.48%) and CHOL (20.29%), with minimal TAG (2.34%), FFA (1.21%), and diacylglycerol (DAG, 1.68%) content. BvE-treated cells displayed a largely similar lipid composition (CHOL EST 75.59%, CHOL 16.61%, TAG 2.15%, FFA 3.21%, DAG 2.45%), indicating that BvE alone does not substantially alter the basal lipid profile. In contrast, PA/OA treatment induced a profound remodeling of the intracellular lipid landscape, with TAG becoming the dominant fraction (27.78%), at the expense of CHOL EST (58.11%) and CHOL (11.93%), reflecting the lipotoxic lipid overload. Notably, co-treatment with PA/OA and BvE partially restored the lipid class distribution towards a profile similar to CTR cells, with a relative reduction in TAG content (23.14%) and a partial recovery of CHOL EST (66.20%) compared to PA/OA alone, while CHOL levels remained low (9.11%). As TLC-based lipid analysis resolves lipid classes at a bulk level and does not provide information on the molecular species composition within each class, including fatty acid chain length, degree of unsaturation, or regioisomeric identity, the observed changes in lipid class distribution can be interpreted as broad alterations in neutral lipid homeostasis rather than precise molecular remodelling of the keratinocyte lipidome. Taken together, these findings demonstrate that PA/OA induces a significant TAG-driven remodeling of the intracellular lipid landscape in HaCaT keratinocytes, and that BvE co-treatment partially counteracts this lipotoxic lipid redistribution by reducing TAG accumulation and partially restoring CHOL ester levels.

### 2.8. BvE Reduces Lipid Accumulation Through the Modulation of the Autophagic–Lysosomal Pathway

To investigate whether the anti-adipogenic effect of BvE is related to changes in the autophagy flux, LD accumulation and PLIN2 expression were studied in the presence of Bafilomycin A1 (BafA1), a well-established autophagy inhibitor that blocks lysosomal acidification and autophagosome–lysosome fusion.

**Figure 5:**
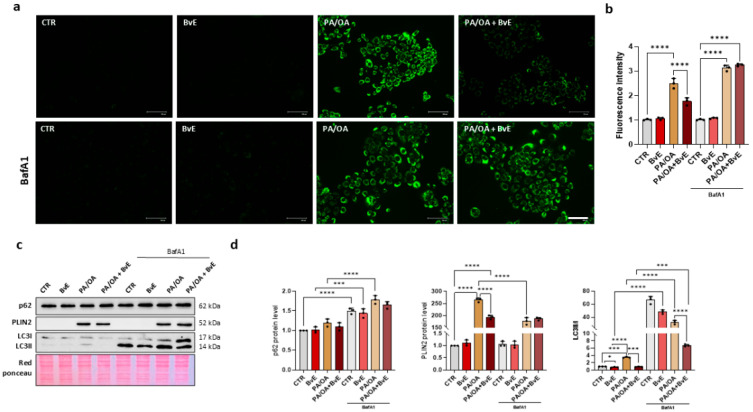
BvE modulates autophagic–lysosomal turnover of lipid droplets under lipotoxic conditions. (**a**) Representative fluorescence images of HaCaT cells treated with *Beta vulgaris* extract (BvE; 1 μg/mL) for 24 h, palmitic acid (PA) and oleic acid (OA) for 48 h (PA/OA; 1:1 ratio, 200 µM each), and pre-treatment of PA/OA for 48 h + BvE for 24 h (co-treatment PA/OA + BvE), in the presence or absence of bafilomycin A1 (BafA1). The green signal indicates lipid droplets (LDs). Scale bars 100 μm. (**b**) Quantification of fluorescence intensity normalized to protein content. (**c**) Western blot analysis of p62, PLIN2 and LC3I/LC3II and (**d**) relative quantification in HaCaT cells treated with BvE (1 μg/mL), PA/OA (1:1 ratio, 200 µM each), and PA/OA + BvE. Red Ponceau staining was used as a total protein loading control. Data are shown as mean ± SD of at least three independent experiments. * *p* < 0.05; *** *p* < 0.005; **** *p* < 0.001.

As shown in Figure 5a,b, in the absence of BafA1, PA/OA treatment significantly increased LD accumulation compared to control cells, as evidenced by enhanced fluorescence intensity. BvE co-treatment markedly reduced LD content in PA/OA-treated cells, restoring fluorescence levels close to those observed in control conditions. Consistently, Western blot analysis, in the absence of BafA1 (Figure 5c,d), revealed that PA/OA treatment induced, compared to control, a significant (**** *p* < 0.001) PLIN2 accumulation. The LC3II/LC3I ratio was increased, compared with the control (*** *p* < 0.005) under this condition, suggesting an autophagic blockade associated with impaired LD turnover.

BvE alone reduced the LC3II/LC3I ratio compared with control cells (* *p* < 0.05), a finding compatible with enhanced autophagic turnover. PA/OA + BvE co-treatment significantly reduced PLIN2 protein levels in PA/OA-treated cells compared with PA/OA alone (*** *p* < 0.005), while further modulating LC3II/LC3I levels in a manner consistent with enhanced autophagic flux. p62 protein level was unchanged (Figure 5c,d).

When autophagy was pharmacologically inhibited by BafA1, the ability of BvE to reduce LD content was substantially abolished, with fluorescence intensity remaining elevated in PA/OA + BvE + BafA1 cells compared to the non-inhibited counterpart (Figure 5a,b). Consistently, the BvE-mediated reduction in PLIN2 protein levels was largely prevented in the presence of BafA1, indicating that PLIN2 degradation induced by BvE is autophagy-dependent (Figure 5c,d). As expected, BafA1 treatment caused accumulation of both LC3II/LC3I and p62 protein levels in BvE, PA/OA and PA/OA + BvE, confirming effective autophagy block.

### 2.9. BvE Suppresses De Novo Lipogenesis and Modulates Key Lipid Metabolism Regulators in PA/OA-Loaded Keratinocytes

**Figure 6:**
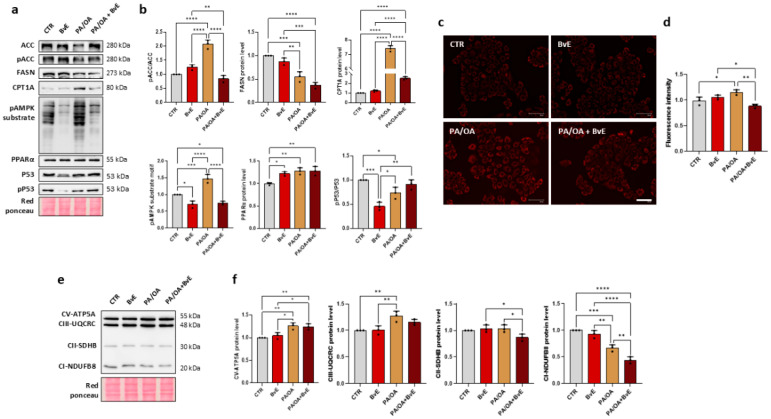
BvE modulates lipogenesis and mitochondrial functions in keratinocytes. (**a**) Western blot analysis of ACC, pACC, FASN, CPT1A, pAMPK substrate, PPARα, P53 and pP53 protein levels and (**b**) relative quantification in HaCaT cells treated with *Beta vulgaris* extract (BvE; 1 μg/mL) for 24 h, palmitic acid (PA) and oleic acid (OA) for 48 h (PA/OA; 1:1 ratio, 200 µM each), and pre-treatment of PA/OA for 48 h + BvE for 24 h (co-treatment PA/OA + BvE). (**c**) Representative fluorescence images showing mitochondrial potential (red; stained with MitoTracker™ Red CMXRos probe) and (**d**) relative fluorescence intensity. Fluorescence intensity was normalized to protein content. (**e**) Western blot analysis of total oxidative phosphorylation (OXPHOS) and (**f**) relative quantification of mitochondrial complex subunits. Ponceau S staining was used as a total protein loading control. Scale bars 100 μm. Data are shown as mean ± SD of at least three independent experiments (* *p* < 0.05; ** *p* < 0.01; *** *p* < 0.005; **** *p* < 0.001).

Given BvE’s ability to regulate lipid accumulation in keratinocytes, its impact on key enzymatic pathways of lipid metabolism was further investigated (Figure 6a,b). The analysis was structured to follow the physiological hierarchy of lipid metabolic regulation, proceeding from *de novo* lipogenesis (DNL) to upstream energy sensing and downstream catabolic programs.

DNL was assessed through two markers: the phosphorylation state of acetyl–CoA carboxylase (ACC) and the expression of fatty acid synthase (FASN). ACC is the rate-limiting enzyme of fatty acid synthesis, whose catalytic activity is allosterically suppressed upon phosphorylation at Serine 79 (pACC-Ser79). FASN catalyzes the terminal step of the DNL pathway.

In PA/OA-treated cells, pACC-Ser79 level was significantly increased (**** *p* < 0.001) and FASN expression was significantly reduced (*** *p* < 0.005) compared to CTR, reflecting a feedback inhibition of endogenous fatty acid synthesis in response to exogenous lipid overload. BvE co-treatment reduced pACC-Ser79 levels compared to PA/OA alone (**** *p* < 0.001), while FASN expression was unchanged compared with PA/OA, indicating that BvE consolidates the inhibition of DNL at multiple enzymatic nodes (Figure 6a,b).

To contextualize these findings within the broader framework of cellular energy sensing, the AMPK signalling pathway was investigated, as AMPK represents the canonical upstream signal system responsible for ACC phosphorylation at Ser79 and a master regulator of the anabolic-to-catabolic metabolic switch.

PA/OA treatment significantly increased the phosphorylation of its downstream targets compared to CTR (*** *p* < 0.005). Notably, both BvE alone and PA/OA + BvE co-treatment significantly reduced AMPK phosphorylation compared to the PA/OA group (**** *p* < 0.001), indicating that BvE attenuates the hyperactivation of this energy-sensing pathway.

Downstream of AMPK, the expression of CPT1A, the rate-limiting enzyme governing the mitochondrial import and subsequent β-oxidation (FAO) of long-chain fatty acids, was markedly upregulated by PA/OA compared to CTR (**** *p* < 0.001). BvE co-treatment significantly antagonized this induction, reducing CPT1A expression compared with PA/OA alone (**** *p* < 0.001).

PPARα, the master transcriptional regulator of CPT1A, was similarly upregulated in both PA/OA and PA/OA + BvE conditions compared to CTR and BvE alone (** *p* < 0.01), further supporting the activation of a PPARα/CPT1A-mediated lipolytic program that BvE does not antagonize but potentially reinforces.

Finally, given the established role of p53 as a multi-stress sensor operating at the intersection of oxidative stress, ER homeostasis, and lipid metabolism, p53 phosphorylation was assessed as an integrative readout of the overall cellular response to BvE treatment. The pP53/P53 ratio was significantly reduced in both BvE-treated (*** *p* < 0.005) and PA/OA-treated cells (* *p* < 0.05) compared to CTR (Figure 6a,b).

### 2.10. BvE Modulates Mitochondrial Functions Under Lipotoxic Conditions

To assess whether AMPK activation paralleled functional changes at the mitochondrial level, mitochondrial membrane potential was evaluated using the MitoTracker™ Red CMXRos probe (Figure 6c). PA/OA-treated cells showed a significant increase in fluorescence intensity compared with CTR (* *p* < 0.05), indicative of mitochondrial hyperpolarization under lipotoxic conditions. At the same time, BvE alone did not significantly alter mitochondrial membrane potential. Strikingly, PA/OA + BvE co-treatment significantly reduced fluorescence intensity compared to PA/OA alone (** *p* < 0.01) and compared to untreated CTR (* *p* < 0.05), demonstrating that BvE attenuates PA/OA-induced mitochondrial alterations (Figure 6d). To further characterize mitochondrial respiratory chain function, the expression of key oxidative phosphorylation (OXPHOS) complex subunits was assessed by Western blot (Figure 6e,f). PA/OA treatment significantly upregulated CV-ATP5A (** *p* < 0.01) and CIII-UQCRC2 (** *p* < 0.01) compared to CTR, with levels partially maintained in the PA/OA + BvE condition. In contrast, CII-SDHB was significantly reduced upon co-treatment compared with PA/OA alone (* *p* < 0.05). Most strikingly, CI-NDUFB8, a subunit of Complex I, was significantly downregulated by PA/OA (** *p* < 0.01) and further reduced in the PA/OA + BvE condition (**** *p* < 0.001).

## 3. Discussion

To our knowledge, this is the first study to investigate the effects of an aqueous *Beta vulgaris* extract (BvE) on lipid metabolism and cellular stress responses in human keratinocytes. We demonstrate that BvE, standardized for betanin content and antioxidant capacity, exerts significant antioxidant, anti-lipogenic, and cytoprotective effects in HaCaT keratinocytes.

NMR metabolite profiling revealed a compositionally rich extract dominated by carbohydrates, glutamine, betanin, betaine, proteinogenic amino acids, and minor components including riboflavin and trigonelline. This complexity is consistent with previously reported profiles of beet-derived aqueous extracts and reflects the high metabolic activity of leaves grown in liquid bioreactor culture [32,33,34,35]. The SETIS^®^ temporary immersion bioreactor system ensures highly standardized and reproducible plant material, a critical prerequisite for mechanistic in vitro studies. Although betanin concentration (0.52 ± 0.09 mg g^−1^ FW) was lower than typically found in commercial beetroot extracts, antioxidant capacity, soluble phenolics content (1.38 ± 0.24 mg GAE g^−1^ FW), and flavonoid concentration (0.32 ± 0.08 mg GAE g^−1^ FW) fell within the range reported for cultivated *B. vulgaris* varieties [19,20,21,22,23,24], confirming the biological relevance of the tested concentrations. Betaine (0.085 ± 0.009 mg ml^−1^), detected alongside betanin and polyphenolic pigments, is of metabolic interest given its established capacity to inhibit hepatic lipogenesis via SREBP-1c and FASN suppression. Its co-presence with other bioactive constituents may underline a multi-component synergy that explains why effects at 1 µg/mL BvE exceed predictions from any single purified constituent [36].

DCFH-DA assay provides a global measure of intracellular ROS accumulation without distinguishing between specific reactive species such as superoxide, hydrogen peroxide, or hydroxyl radicals. By using this probe, we detected a significant reduction by BvE in oxidative burden in H_2_O_2_-exposed HaCaT cells. This was accompanied by elevated NRF2 protein levels even in the absence of exogenous stress, suggesting intrinsic activation of the KEAP1-NRF2 pathway, consistent with previously reported betanin-mediated NRF2 nuclear translocation in neuronal and hepatic models [37]. The observed downregulation of catalase and GPx-4 under basal BvE treatment likely reflects reduced cellular demand for enzymatic antioxidant activity, a homeostatic adjustment consistent with the oxidative stress compensation model, rather than impairment of antioxidant defence. Under H_2_O_2_ co-treatment, the strong suppression of GPx-4 alongside maintained NRF2 elevation suggests that BvE provides upstream chemical interception of lipid-peroxidizing species, reducing the substrate load for GPx-4 and thus the physiological necessity for its induction. This is consistent with the established capacity of betalains to directly quench lipid peroxyl radicals [33,38]. Unchanged SOD1 levels across all conditions confirm that the response is specific to the peroxidative cascade [39].

The central finding of this study is the marked attenuation of PA/OA-induced LD accumulation, quantified by BODIPY fluorescence, accompanied by mechanistically coherent changes in LD-regulatory protein expression. DGAT1, which catalyzes the committed rate-limiting step of TAG biosynthesis, was upregulated by PA/OA and partially reversed by BvE, suggesting suppression of terminal lipogenic flux [40,41]. The concomitant reduction in ATGL and MAGL, enzymes canonically associated with LD mobilization, is interpreted in the context of a global attenuation of lipid metabolic flux: by reducing LD biogenesis upstream via DGAT1 suppression, BvE likely diminishes the substrate availability that drives compensatory lipolytic upregulation [42].

PLIN2, the dominant structural coat protein of LDs in non-adipose cells [43], was massively induced by PA/OA and substantially reduced by BvE co-treatment, coherent with the reduction in LD accumulation. Inhibition of autophagy by BafA1 abolished the BvE-mediated reduction in both LD and PLIN2 protein levels, providing direct evidence that the extract exerts its lipid-lowering effect through an autophagy-dependent mechanism.

TLC-based lipid class analysis confirmed a profound PA/OA-induced remodelling of the neutral lipid landscape, elevating TAG from ~2% to ~28% of total neutral lipids at the expense of cholesterol esters. BvE co-treatment partially reversed this pattern, reducing TAG to ~23% and partially restoring cholesterol ester content to ~66%, indicating a preferential effect on TAG metabolism. The residual TAG elevation likely reflects the kinetics of experimental design, as BvE was administered only during the final 24 h of a 48-h PA/OA exposure, providing a therapeutic rather than preventive window. Nonetheless, the ~16% relative reduction in TAG content within this constrained timeframe argues for an acute modulatory effect on TAG turnover.

AMPK was robustly activated by PA/OA, indicating activation of a catabolic response. AMPK simultaneously inactivates ACC, the rate-limiting enzyme of DNL, and upregulated CPT1A [44]. Consequently, FASN was progressively downregulated by PA/OA and further suppressed by BvE, with AMPK-mediated ACC inhibition depleting the malonyl–CoA substrate pool. The coordinated upregulation of PPARα and CPT1A in PA/OA and PA/OA + BvE conditions further supports induction of a compensatory mitochondrial catabolic program, which BvE does not antagonize.

The AMPK axis has been extensively characterized in metabolically active non-epidermal cell types. In hepatocytes and adipocytes, AMPK activation promotes phosphorylation of PLIN2 at Ser492, targeting it for chaperone-mediated autophagic degradation and restoring lipolytic access to the LD core [8]. In skeletal muscle, a comparable mechanism has been linked to exercise-induced lipid mobilisation. However, whether this axis is functionally operative in keratinocytes has not been previously demonstrated. The present findings showing a concomitant reduction in AMPK signaling and PLIN2 protein levels following BvE treatment under lipotoxic conditions.

Although AMPK and PLIN2 were both upregulated under PA/OA-induced lipotoxic conditions, this co-elevation likely reflects a state of metabolic overload in which the transcriptional induction of PLIN2, driven by the massive accumulation of neutral lipids [45], overwhelms the capacity of AMPK-mediated phosphorylation to target PLIN2 for chaperone-mediated autophagic degradation. Conversely, BvE co-treatment reduced both AMPK activation and PLIN2 protein levels, suggesting that BvE acts upstream of this axis by attenuating LD biogenesis, thereby diminishing the primary stimulus for PLIN2 transcriptional induction rather than directly modulating the AMPK-PLIN2 degradation axis itself.

BvE also induced a broad suppression of all three canonical UPR branches (IRE1α/XBP1s, PERK/eIF2α, and ATF6) and the master ER chaperone GRP78/BiP in HaCaT cells. Simultaneous downregulation across all branches suggests reduction in the global ER proteostatic load rather than selective antagonism of a single sensor, consistent with reduced lipid-induced bilayer stress, a recognized primary trigger for IRE1α and PERK oligomerization. Downregulation of GRP78 should be interpreted because of diminished ER stress rather than reduced ER folding capacity. These effects were reproduced in HCT15 intestinal epithelial cells, extending the cytoprotective profile of BvE across the epithelial lineage [46,47].

At the mitochondrial level, PA/OA induced significant hyperpolarization of mitochondrial membrane potential (ΔΨm), an early pathophysiological indicator of lipotoxic dysfunction reflecting over-reduction in the electron transport chain (ETC) and consequent ROS generation. BvE co-treatment significantly attenuated this hyperpolarization, suggesting a protective effect on mitochondrial bioenergetics. OXPHOS subunit analysis revealed selective downregulation of CI-NDUFB8 by PA/OA, further accentuated by BvE, alongside significant reduction in CII-SDHB in the combined condition, suggesting a broader mitochondrial remodelling consistent with hormetic mitochondrial adaptation. Furthermore, although direct functional assays like Seahorse respirometry or ATP measurements were not performed, the convergent evidence from ΔΨm quantification and OXPHOS profiling provides a consistent overview of BvE’s mitochondrial effects, establishing a solid baseline for future functional investigations.

Finally, the progressive downregulation of the pP53/P53 ratio across treatment conditions provides an integrative readout of the cellular stress landscape. The broad suppression of UPR signalling by BvE would be expected to reduce PERK-mediated p53 phosphorylation, consistent with resolved ER stress, while AMPK-mediated FASN repression may provide an additional transcriptional brake on lipogenesis via p53-dependent mechanisms.

These observed biological effects correlate well with the comprehensive chemical profile of BvE outlined in Figure 2. Specifically, the high abundance of sugars, alongside specific lipotropic compounds like betaine, provides a clear functional and chemical rationale for the subsequent molecular modifications, directly linking the extract’s composition to the mitigation of cellular stress and lipid dysregulation. 

## 4. Materials and Methods

### 4.1. Seed Sterilization, Pre-Germination, and TIBs SETIS™ Seedling Growth

*Beta vulgaris* L. subsp. vulgaris Beetroot Group ‘Aplastada de Egipto’ seeds were sterilized in a desiccator using chlorine gas [48] with slight modifications; 35 mL of 5% titrated bleach (Niclor 5, OGNA, Milan, Italy) and 4 mL of 34% HCl were used to generate chlorine gas, and the seeds were exposed to it for 20 min. After exposure, the chlorine gas was removed by activating a vacuum pump. The sterile seeds were vernalized at 4 °C for 48 h and germinated on Petri dishes containing sterile solid MS medium pH 5.75, including 2.2 g/L MS, 10 g/L sucrose, and 10 g/L agar (Duchefa Biochemie, Haarlem, The Netherlands). Seedlings were incubated in a growth chamber with 22 ± 1 °C, 70–90 µmol m ^−2^ s ^−1^ light flux and a 16-h light/8-h dark photoperiod. Once germinated, the seedlings were transferred into TIBs (Temporary Immersion Bioreactors) SETIS™ (Vervit, Lochristi, Belgium) filled with sterile MS medium pH 5.75 containing 4.4 g/L MS and 15 g/L sucrose (Duchefa Biochemie, Haarlem, The Netherlands) with Plant Preservative Mixture (PPM, Plant Cell Technology, Washington, DC, USA) at 0.1% (*v*/*v*). The TIBs were maintained in the same growth chamber with identical conditions as during germination. Seedlings were submerged in the liquid medium for 2 min every 6 h [49,50].

### 4.2. Leaf Harvesting and BvE Preparation, and Quantification

Long-term culture in TIBs was established from 30-day plants grown in TIBs. These were harvested and sectioned under sterile conditions into roots (which were eliminated), leaves (useful for extract preparation), and vegetative apexes (1–2 cm), which were transferred to a new TIBs SETIS™ containing a fresh growing medium to restart biomass production in 30-day cycles. The leaves collected every 30 days were placed in 50 mL Falcon tubes, freeze-dried, and stored at −80 °C. The FW and the DW of the material in each Falcon tube were recorded. BvE was obtained in water, used as an extraction buffer due to its superior ability to recover betalains from plant tissues [51]. The extraction procedure consisted of two steps: first, the material was rehydrated to its corresponding fresh weight; subsequently, additional water was added to reach a final fresh weight-to-liquid ratio of 1:2. The extracts were obtained by grinding the leaves with water in a chilled glass mortar. The mixture was centrifuged at 4500 rpm (3900× *g*) for 10 min at 4 °C. The supernatant was collected, divided into 1 mL aliquots, and stored at −80 °C.

Betanin concentration was determined using the Lambert–Beer equation by measuring absorbance at 538 nm, the characteristic wavelength for betacyanin, with betanin being one of the most abundant pigments. A molar extinction coefficient of 60,000 and a molecular weight of 550 g/mol were employed for calculations [33,51,52]. Spectrophotometric measurements were performed using the UV2600 spectrophotometer (Shimadzu, Kyoto, Japan). Betanin concentration is reported as the mean and standard deviation across three independent batches.

### 4.3. Determination of BvE Soluble Phenolics Content

The soluble phenolics content of the BvE was quantified utilizing the Folin–Ciocalteu assay [53] with slight modifications. Specifically, 5 µL of BvE was diluted to a final volume of 50 µL with RPE water, followed by the addition of 450 µL of RPE water and 50 µL of Folin–Ciocalteu reagent, with thorough mixing. After a five-minute incubation, 500 µL of 7% Na_2_CO_3_ solution and 200 µL of RPE water were added, bringing the final volume to 1250 µL, and the samples were mixed again. For the blank, 5 µL of RPE water replaced the BvE. The samples were then incubated in darkness at 20 °C for 90 min, and absorbance was measured at 750 nm. Phenol concentration was determined via a calibration curve constructed with gallic acid standards (10, 20, 40, 80, 100, 120 µg/mL) prepared in RPE water. Spectrophotometric readings were acquired using a Shimadzu UV2600 spectrophotometer (Kyoto, Japan). Phenols concentration is reported as the mean and standard deviation across three independent batches.

### 4.4. Determination of BvE Flavonoid Content

The flavonoid content of the BvE was quantified following the method described in [53], with slight modifications. A volume of 10 µL of BvE was diluted to 500 µL with RPE water. Subsequently, 30 µL of 5% NaNO_2_ solution was added, and the mixture was homogenized. After 5 min at 20 °C, 60 µL of 10% AlCl_3_ solution was incorporated. The samples were mixed again, and after 6 min, 200 µL of 1 M NaOH and 210 µL of RPE water were added, bringing the final volume to 1000 µL. The mixture was thoroughly mixed. A blank was prepared by replacing BvE with 10 µL of RPE water. Absorbance measurements were taken at 510 nm using a UV2600 spectrophotometer (Shimadzu, Kyoto, Japan). A calibration curve was established with known concentrations of catechin standard (3.125, 6.25, 12.5, 25, 50, 100, 200, 400 µg/mL) in RPE water. Flavonoid concentration is reported as mean and standard deviation across three independent batches.

### 4.5. Determination of BvE Nitrate Content

The nitrate content of the BvE was determined following the method outlined in [54], with some modifications. A 5 µL aliquot of BvE was diluted to a final volume of 25 µL with RPE water. To this, 80 µL of 5% salicylic acid solution prepared in concentrated H_2_SO_4_ was added, and the samples were mixed. After incubation for 20 min at 20 °C, 950 µL of RPE water was added, and the mixture was briefly mixed before adding 950 µL of 4 M NaOH. The samples were mixed again. Due to the pigmentation of BvE, blanks were prepared individually for each sample using a 5 µL aliquot of BvE, but substituting the salicylic acid solution with only concentrated H_2_SO_4_. Following centrifugation at 13,000 rpm (15,500× *g*) for 1 min, absorbance was measured at 410 nm. A calibration curve was constructed using known concentrations of NO_3_-N (20, 40, 60, 80, 100, 120 µg/mL) to quantify nitrate levels. The NO_3_-N stock solution (0.500 µg/mL) was prepared by dissolving 0.1805 g of KNO_3_ in 50 mL of RPE water. Results were expressed as NO_3_–N and converted to NO_3_^−^ using the molecular weight ratio NO_3_^−^/N = 4.43. Spectrophotometric measurements were performed on a UV2600 spectrophotometer (Shimadzu, Kyoto, Japan). Nitrate concentration is reported as mean and standard deviation across three independent batches.

### 4.6. Determination of BvE Antioxidant Activity

The determination of antioxidant activity was carried out using the TEAC (Trolox Equivalent Antioxidant Capacity) assay [55] with slight modifications. The ABTS·^+^ solution was diluted 1:90 in 5 mM Phosphate-Buffered Saline (PBS), pH 7.4. Three aliquots of 10 µL of each BvE were transferred into 2 mL microcentrifuge tubes, and 1 mL of diluted ABTS·^+^ solution was added for the determination of hydrophilic antioxidant capacity. Control samples (0 µM Trolox) were prepared by adding 10 µL of methanol to 1 mL of ABTS·^+^ solution, while the blank consisted of 1 mL of PBS. The reaction mixtures were incubated for 15 min at 20 °C in the dark, after which they were transferred into cuvettes and the absorbance was measured at 734 nm. A linear calibration curve ranging from 0 to 15 µM Trolox was prepared in PBS for quantifying antioxidant activity. Spectrophotometric measurements were performed using the UV2600 spectrophotometer (Shimadzu, Kyoto, Japan). The antioxidant capacity of BvE was expressed as Trolox equivalents (TE) and reported as µmol TE mL^−1^ and µmol TE g^−1^. Antioxidant capacity is reported as the mean and standard deviation across three independent batches.

### 4.7. NMR Analysis of BvE 

BvE was lyophilized and redissolved in the same volume of deuterium oxide (D_2_O) (99.9 atom %D) containing 0.05% wt 3-(trimethylsilyl)propionic-2,2,3,3 d4 acid sodium salt (TSP) (Sigma-Aldrich, St. Louis, MO, USA). Then, samples were spun down in a microcentrifuge at 10,000 g for 10 min at 4 °C, and the supernatants were transferred to 5 mm NMR tubes. All measurements were performed on a Bruker Avance III 600 Ascend NMR spectrometer (Bruker, Ettlingen, Germany), operating at 600.13 MHz for ^1^H observation, equipped with a TCI cryoprobe (triple resonance inverse cryoprobe) incorporating a z-axis gradient coil and automatic tuning matching (ATM). Experiments were acquired at 300 K. For each sample a 1D sequence with pre-saturation and composite pulse for selection (zgcppr Bruker standard pulse sequence) was acquired, with 128 transients, 16 dummy scans, 5 s relaxation delay, a size of Free Induction Decay (FID) of 64K data points, a spectral width of 12,019.230 Hz (20.0276 ppm), and an acquisition time of 2.73 s. Spectra were processed in a standardized manner using Topspin 4.1.4 (Bruker, Biospin, Italy) with 0.3 Hz line-broadening, Fourier transformation, phasing, and baseline correction [56]. Metabolite identifications were based on ^1^H (^1^H-^1^H J-resolved, ^1^H-^1^H COSY correlation spectroscopy) and ^13^C (^1^H-^13^C heteronuclear single quantum correlation, HSQC; ^1^H−^13^C heteronuclear multiple bond correlation, HMBC) assignment by 1D and 2D omo- and heteronuclear experiments and by comparison with literature data. Quantification of metabolites was performed using the ratio method [29]; TSP (δ = 0.00 ppm) was used as an internal standard. The compositional reproducibility of BvE was evaluated by analyzing three independent batches.

### 4.8. Cell Treatments and Viability Test

Human colon cancer cell line HCT15 and human immortalized keratinocytes HaCaT were maintained in Dulbecco’s Modified Eagle Medium (DMEM, D5546, Sigma-Aldrich, St. Louis, MO, USA) supplemented with 10% fetal bovine serum (FBS, Capricorn HI-12A), 100 U/mL penicillin, 100 μg/mL streptomycin (#P4333, Sigma-Aldrich, St. Louis, MO, USA), and 2 mM glutamine (G7513, Sigma-Aldrich, St. Louis, MO, USA). Cells were cultured at 37 °C in a humidified atmosphere with 5% CO_2_ and regularly screened for mycoplasma contamination using a Mycoplasma Detection Kit (Aurogene, Roma, Italy, REP-MYSNC-100).

To induce lipid accumulation and metabolic stress, cells were exposed to a mixture of PA (COD. 506345) and OA (COD. 75090) (Sigma-Aldrich, St. Louis, MO, USA). Fatty acids were dissolved in fatty-acid-free bovine serum albumin (BSA, COD. 03117057001) at 8 mM concentration and were subsequently diluted to the desired final concentration in DMEM immediately before cell treatments. For cell treatments with PA and OA, we chose the concentration of 0.2 mM, which represents the physiological postprandial intestinal concentration [57,58] and is within the concentration range of FFAs in human plasma (i.e., 0.2–2 mM) [58,59]. To evaluate the therapeutic potential of BvE, the treatment was administered in a post-insult phase: after the first 24 h of PA/OA exposure, the medium was supplemented with BvE (1 µg/mL) for the remaining 24 h of the experiment. For oxidative stress induction, hydrogen peroxide (H_2_O_2_, COD. 216763) was used as a positive control at a final concentration of 400 µM for 24 h. For lysosomal degradation inhibition, BafA1 (COD. 54645s, Santa Cruz Biotechnology, Dallas, TX, USA) was used as positive control at a final concentration of 200 nM, added 3 h before the end of the treatment.

For viability assay, HCT15 and HaCaT cells were seeded at 5 × 10^4^ cells/well in 96-well plates in DMEM supplemented with 10% FBS and 1% antibiotics. After treatment, Alamar Blue reagent was added. The plate was incubated for 4 h at 37 °C in a humidified atmosphere with 5% CO_2_. Fluorescence was read at 560/590 nm by a Biotek Cytation 5 Cell Imaging Multimode Reader (Agilent Technologies, Santa Clara, CA, USA).

### 4.9. Fluorimetric Assessments

Intracellular ROS level was assessed using the cell-permeable fluorescent probe 2′,7′-dichlorodihydrofluorescein diacetate (DCFH-DA, D399). HCT15 and HaCaT cells were seeded in 12-well plates. After treatments, cells were washed with PBS and incubated with a fresh serum-free culture medium containing 20 μM DCFH-DA for 30 min at 37 °C, in the dark. Following incubation, cells were washed twice with PBS to remove the extracellular dye and resuspended in PBS for imaging. Images were captured using a fluorescence microscope (Cell Imaging Station Microscope, Invitrogen™, FLoid™ Cell Imaging Station, 10 213–412, ThermoFisher Scientific, Waltham, MA, USA) at 20× magnification with a scale bar of 100 μm. The mean fluorescence intensity, proportional to ROS production, was quantified using ImageJ 1.54 software (NIH, Bethesda, MD, USA). Fluorescence intensity was measured at 485/530 nm by a Biotek Cytation 5 Cell Imaging Multimode Reader (Agilent Technologies, Santa Clara, CA, USA) and normalized to protein content.

For the BODIPY stain protocol, used for LD analysis, cells were seeded onto coverslips in 12-well plates at 60–80% confluence. After treatment, cells were washed twice with PBS and incubated for 15 min at 37 °C with BODIPY 493/503 at a dilution of 1:1000 in PBS from a 1 mg/mL stock solution. Thereafter, cells were washed three times with PBS and used for rapid image acquisition. Images were captured using a Cell Imaging Station microscope (Invitrogen™, FLoid™ Cell Imaging Station, 10-213-412, ThermoFisher Scientific, Waltham, MA, USA) at 20× magnification with a scale bar of 100 μm. Fluorescence intensity was measured at 485/530 nm by a Biotek Cytation 5 Cell Imaging Multimode Reader (Agilent Technologies, Santa Clara, CA, USA) and normalized to protein content.

Mitochondrial membrane potential was evaluated using the MitoTracker™ Red CMXRos fluorescent probe (ThermoFisher Scientific, #M7512). Following the experimental treatments, HCT15 and HaCaT cells seeded in 12-well plates were washed twice with PBS to remove serum traces and metabolic byproducts. Cells were then incubated with a 100 nM MitoTracker™ Red solution, diluted in serum-free medium or PBS, for 20 min at 37 °C in a humidified atmosphere with 5% CO_2_. To minimize background fluorescence, cells were washed again with PBS after incubation. For quantitative assessment, the fluorescence intensity was measured at 570/620 nm by a Biotek Cytation 5 Cell Imaging Multimode Reader (Agilent Technologies, Santa Clara, CA, USA) and normalized to protein content. Images were captured using a Cell Imaging Station microscope (Invitrogen™, FLoid™ Cell Imaging Station, 10 213412, ThermoFisher Scientific) at 20× magnification with a scale bar of 100 μm.

### 4.10. TLC Analysis of Lipids

Total lipids were extracted from HaCaT and HCT15 cells using methyl-tert-butyl ether as an extraction solvent, as reported in [60]. Extracted lipids were loaded on silica gel plates for TLC analysis. Plates were developed with hexane/ethyl ether/acetic acid (70/30/1; *v*/*v*/*v*). After development, plates were uniformly sprayed with 10% cupric sulfate in 8% aqueous phosphoric acid, allowed to dry for 10 min at room temperature, and then placed into a 145 °C oven for 10 min, as reported in [61]. Different lipid species were identified by developing specific standards under the same experimental conditions. Spot intensity was measured by densitometric analysis.

### 4.11. Western Blot Analyses

Cellular proteins were extracted from cells using RIPA lysis buffer (Cell signaling Technology, Danvers, MA, USA, #9806). Total protein level was determined using the Bradford method (Bio-Rad Laboratories, Hercules, CA, USA, Cat. no 5000201). After boiling for 5 min, proteins were loaded and separated by SDS polyacrylamide gel electrophoresis. The samples were then transferred onto a nitrocellulose membrane (Bio-Rad Laboratories, Hercules, CA, USA, Cat. no 1620115) and blocked at room temperature for 1 h using 5% (*w*/*v*) non-fat milk in TBS-Tris buffer (Tris-buffered saline (TBS) plus 0.5% (*v*/*v*) Tween−20, TTBS). The membranes were incubated with the primary antibodies (Table S2). After washing with TTBS, the blots were incubated with peroxidase-conjugated monoclonal secondary antibodies (Cell signaling Technology, Danvers, MA, USA, #7074 or #7076s) at 1:10,000 dilutions at room temperature for 1–2 h. The blots were then washed thoroughly in TTBS. Western blotting analyses were performed using the Amersham ECL Advance Western Blotting Detection Kit (GE Healthcare, Little ChaOEAnt, UK), and the ChemiDoc system (Bio-Rad Laboratories, Hercules, CA, USA, Cat. no 12003153) was used for 6 chemiluminescence measurements. Densitometric analysis of the immunoblots was performed using Image LabTM Version 6.0.1 2017 (Bio-Rad Laboratories, Hercules, CA, USA) software. 

### 4.12. Statistical Analysis

All experiments were performed in at least three independent biological replicates. Data are expressed as mean ± standard deviation (SD). Given the nature of the data and the number of replicates, parametric testing was applied assuming approximate normality. Statistical analysis was performed using GraphPad Prism 9.1.0. Comparisons between two groups were made using a two-tailed Student’s t-test. Multiple group comparisons were performed by one-way analysis of variance (ANOVA); where all pairwise comparisons were of interest, ANOVA was followed by Tukey’s honestly significant difference (HSD) post hoc test. A *p*-value < 0.05 was considered statistically significant.

## 5. Conclusions

In conclusion, BvE exerts coordinated antioxidant, anti-lipogenic, ER stress-resolving, and mitochondria-protective effects in human keratinocytes through a mechanistically integrated network engaging the KEAP1-NRF2, AMPK-ACC-FASN, and UPR axes. These findings are relevant in the context of keratinocyte lipid dysregulation underlying skin pathologies such as obesity-associated cutaneous metabolic dysfunction. In addition, it must be acknowledged that the present study did not include fractionation of BvE or direct comparison with isolated constituents at equivalent molar concentrations. Consequently, the relative contributions of individual components, and potential synergistic or antagonistic interactions among them, cannot be determined from the current dataset. Future studies employing activity-guided fractionation and testing of purified compounds will be necessary to deconvolute the molecular basis of the observed effects. Moreover, our results are derived from a two-dimensional monoculture model of immortalized keratinocytes, and translational implications should be regarded as exploratory hypotheses requiring validation in three-dimensional skin equivalents and appropriately designed clinical studies. Therefore, future investigations will focus on expanding the extract’s chemical characterization via LC-MS, benchmarking its efficacy against pure bioactive standards and field-grown counterparts, and translating these mechanistic in vitro findings into appropriate in vivo animal models.

## Figures and Tables

**Table 1:** Phytochemical profile and antioxidant activity of the extract.

Compounds	mL^−1^	g^−1^ FW
Betanin (mg)	0.24 ± 0.03	0.52 ± 0.09
Soluble phenol (mg GAE)	0.69 ± 0.12	1.38 ± 0.24
Flavonoids (mg CE)	0.16 ± 0.04	0.33 ± 0.08
Nitrate (mg NO_3_^−^)	0.96 ±0.35	1.92 ± 0.70
Antioxidant activity (µmol TE)	4.75 ± 1.14	10.08 ± 2.50

## Data Availability

All experimental data are provided in the manuscript or Supplementary Materials.

## Supplementary Materials

The following supporting information can be downloaded at https://www.mdpi.com/article/10.3390/ijms27114816/s1.

## Abbreviations

The following abbreviations are used in this manuscript:

ACAT	Acyl–CoA:cholesterol acyltransferase
ACC	Acetyl–CoA carboxylase
ARE	Antioxidant response element
ATGL	Adipose triglyceride lipase
ATM	Automatic tuning matching
BSA	Fatty-acid-free bovine serum albumin
BvE	*Beta vulgaris* extract
CHOL	Cholesterol
CHOL EST	Cholesterol esters
CMA	Chaperone-mediated autophagy
CTR	Control
D_2_O	Deuterium oxide
DAG	Diacylglycerol
DCFH-DA	2′,7′-dichlorodihydrofluorescein diacetate
DGAT1	Diacylglycerol O-acyltransferase 1
DNL	*De novo* lipogenesis
DW	Dry weight
E-FABP	Epidermal fatty acid binding protein
ER	Endoplasmic reticulum
ETC	Electron transport chain
FAO	Fatty acid β-oxidation
FFA	Free fatty acids
FW	Fresh weight
GPATs	Glycerol−3-phosphate acyltransferases
H_2_O_2_	Hydrogen peroxide
^1^H NMR	Proton nuclear magnetic resonance
LDs	Lipid droplets
MAGL	Monoacylglycerol lipase
OA	Oleic acid
OXPHOS	Oxidative phosphorylation
PA	Palmitic acid
PLIN2	Perilipin 2
ROS	Reactive oxygen species
TAG	Triacylglycerol
TE	Trolox equivalents
TEAC	Trolox Equivalent Antioxidant Capacity
TIBs	Temporary Immersion Bioreactors
TLC	Thin-layer chromatography
UPR	Unfolded protein response

## References

[1] Gruber F., Marchetti-Deschmann M., Kremslehner C., Schosserer M. (2021). The Skin Epilipidome in Stress, Aging, and Inflammation. Front. Endocrinol..

[2] Elias P.M., Williams M.L., Holleran W.M., Jiang Y.J., Schmuth M. (2008). Thematic Review Series: Skin Lipids. Pathogenesis of Permeability Barrier Abnormalities in the Ichthyoses: Inherited Disorders of Lipid Metabolism. J. Lipid Res..

[3] Walther T.C., Farese R.V. (2012). Lipid Droplets and Cellular Lipid Metabolism. Annu. Rev. Biochem..

[4] Thiam A.R., Farese R.V., Walther T.C. (2013). The Biophysics and Cell Biology of Lipid Droplets. Nat. Rev. Mol. Cell Biol..

[5] Pang B., Zhu Z., Xiao C., Luo Y., Fang H., Bai Y., Sun Z., Ma J., Dang E., Wang G. (2022). Keratin 17 Is Required for Lipid Metabolism in Keratinocytes and Benefits Epidermal Permeability Barrier Homeostasis. Front. Cell Dev. Biol..

[6] Loix M., Wouters E., Vanherle S., Dehairs J., McManaman J.L., Kemps H., Swinnen J.V., Haidar M., Bogie J.F.J., Hendriks J.J.A. (2022). Perilipin−2 Limits Remyelination by Preventing Lipid Droplet Degradation. Cell. Mol. Life Sci..

[7] Corsini E., Viviani B., Zancanella O., Lucchi L., Bartesaghi S., Galli C.L., Marinovich M., Visioli F., Serrero G. (2003). Induction of Adipose Differentiation Related Protein and Neutral Lipid Droplet Accumulation in Keratinocytes by Skin Irritants. J. Investig. Dermatol..

[8] Kaushik S., Cuervo A.M. (2016). AMPK-Dependent Phosphorylation of Lipid Droplet Protein PLIN2 Triggers Its Degradation by CMA. Autophagy.

[9] Ertunc M.E., Hotamisligil G.S. (2016). Lipid Signaling and Lipotoxicity in Metaflammation: Indications for Metabolic Disease Pathogenesis and Treatment. J. Lipid Res..

[10] Pavlov A., Bley T. (2006). Betalains Biosynthesis by *Beta vulgaris* L. Hairy Root Culture in a Temporary Immersion Cultivation System. Process Biochem..

[11] Zhang Y., Li Q., Rao E., Sun Y., Grossmann M.E., Morris R.J., Cleary M.P., Li B. (2015). Epidermal Fatty Acid Binding Protein Promotes Skin Inflammation Induced by High-Fat Diet. Immunity.

[12] Kubo A. (2014). Nagashima-Type Palmoplantar Keratosis: A Common Asian Type Caused by SERPINB7 Protease Inhibitor Deficiency. J. Investig. Dermatol..

[13] Zaccaron R.P., Mendes C., da Costa C., Silveira P.C.L., Rezin G.T. (2024). Skin Metabolism in Obesity: A Narrative Review. Wound Repair Regen..

[14] Yew Y.W., Mina T., Ng H.K., Lam B.C.C., Riboli E., Lee E.S., Lee J., Ngeow J., Elliott P., Thng S.T.G. (2023). Investigating Causal Relationships between Obesity and Skin Barrier Function in a Multi-Ethnic Asian General Population Cohort. Int. J. Obes..

[15] Ikeda K., Morizane S., Akagi T., Hiramatsu-Asano S., Tachibana K., Yahagi A., Iseki M., Kaneto H., Wada J., Ishihara K. (2022). Obesity and Dyslipidemia Synergistically Exacerbate Psoriatic Skin Inflammation. Int. J. Mol. Sci..

[16] Vulić J.J., Ćebović T.N., Čanadanović-Brunet J.M., Ćetković G.S., Čanadanović V.M., Djilas S.M., Tumbas Šaponjac V.T. (2014). In Vivo and in Vitro Antioxidant Effects of Beetroot Pomace Extracts. J. Funct. Foods.

[17] Gandía-Herrero F., Escribano J., García-Carmona F. (2016). Biological Activities of Plant Pigments Betalains. Crit. Rev. Food Sci. Nutr..

[18] Song Z., Deaciuc I., Zhou Z., Song M., Chen T., Hill D., McClain C.J. (2007). Involvement of AMP-Activated Protein Kinase in Beneficial Effects of Betaine on High-Sucrose Diet-Induced Hepatic Steatosis. Am. J. Physiol.-Gastrointest. Liver Physiol..

[19] Chhikara N., Kushwaha K., Sharma P., Gat Y., Panghal A. (2019). Bioactive Compounds of Beetroot and Utilization in Food Processing Industry: A Critical Review. Food Chem..

[20] Ramírez-Melo L.M., Cruz-Cansino N.d.S., Delgado-Olivares L., Ramírez-Moreno E., Zafra-Rojas Q.Y., Hernández-Traspeña J.L., Suárez-Jacobo Á. (2022). Optimization of Antioxidant Activity Properties of a Thermosonicated Beetroot (*Beta vulgaris* L.) Juice and Further in Vitro Bioaccessibility Comparison with Thermal Treatments. LWT.

[21] Trych U., Buniowska-Olejnik M., Marszałek K. (2022). Bioaccessibility of Betalains in Beetroot (*Beta vulgaris* L.) Juice under Different High-Pressure Techniques. Molecules.

[22] Sokolova D.V., Shvachko N.A., Mikhailova A.S., Popov V.S., Solovyeva A.E., Khlestkina E.K. (2024). Characterization of Betalain Content and Antioxidant Activity Variation Dynamics in Table Beets (*Beta vulgaris* L.) with Differently Colored Roots. Agronomy.

[23] Barba-Espin G., Glied-Olsen S., Dzhanfezova T., Joernsgaard B., Lütken H., Müller R. (2018). Preharvest Application of Ethephon and Postharvest UV-B Radiation Improve Quality Traits of Beetroot (*Beta vulgaris* L. ssp. Vulgaris) as Source of Colourant. BMC Plant Biol..

[24] Takács-Hájos M., Vargas-Rubóczki T. (2022). Evaluation of Bioactive Compounds in Leaf and Root of Five Beetroot Varieties. J. Agric. Food Res..

[25] Grzebelus D., Baranski R. (2001). Identification of Accessions Showing Low Nitrate Accumulation in a Germplasm Collection of Garden Beet. Acta Hortic..

[26] Bescos R., Rollason M.L., Davies T.S., Casas-Agustench P. (2023). Content of Nitrate and Nitrite in Commercial and Self-made Beetroot Juices and the Effect of Storage Temperature. Food Sci. Nutr..

[27] Iammarino M., Berardi G., Vita V., Elia A., Conversa G., Di Taranto A. (2022). Determination of Nitrate and Nitrite in Swiss Chard (*Beta vulgaris* L. subsp. Vulgaris) and Wild Rocket (*Diplotaxis tenuifolia* (L.) DC.) and Food Safety Evaluations. Foods.

[28] Kostidis S., Addie R.D., Morreau H., Mayboroda O.A., Giera M. (2017). Quantitative NMR Analysis of Intra- and Extracellular Metabolism of Mammalian Cells: A Tutorial. Anal. Chim. Acta.

[29] Gogna N., Hamid N., Dorai K. (2015). Metabolomic Profiling of the Phytomedicinal Constituents of *Carica papaya* L. Leaves and Seeds by 1H NMR Spectroscopy and Multivariate Statistical Analysis. J. Pharm. Biomed. Anal..

[30] Giampaoli O., Sciubba F., Conta G., Capuani G., Tomassini A., Giorgi G., Brasili E., Aureli W., Miccheli A. (2021). Red Beetroot’s NMR-Based Metabolomics: Phytochemical Profile Related to Development Time and Production Year. Foods.

[31] Moliterni C., Vari F., Schifano E., Tacconi S., Stanca E., Friuli M., Longo S., Conte M., Salvioli S., Gnocchi D. (2024). Lipotoxicity of Palmitic Acid Is Associated with DGAT1 Downregulation and Abolished by PPARα Activation in Liver Cells. J. Lipid Res..

[32] Georgiev V.G., Weber J., Kneschke E.-M., Denev P.N., Bley T., Pavlov A.I. (2010). Antioxidant Activity and Phenolic Content of Betalain Extracts from Intact Plants and Hairy Root Cultures of the Red Beetroot *Beta vulgaris* Cv. Detroit Dark Red. Plant Foods Hum. Nutr..

[33] Stintzing F.C., Carle R. (2004). Functional Properties of Anthocyanins and Betalains in Plants, Food, and in Human Nutrition. Trends Food Sci. Technol..

[34] De Carlo A., Tarraf W., Lambardi M., Benelli C. (2021). Temporary Immersion System for Production of Biomass and Bioactive Compounds from Medicinal Plants. Agronomy.

[35] Ozyigit I.I., Dogan I., Hocaoglu-Ozyigit A., Yalcin B., Erdogan A., Yalcin I.E., Cabi E., Kaya Y. (2023). Production of Secondary Metabolites Using Tissue Culture-Based Biotechnological Applications. Front. Plant Sci..

[36] Williamson E. (2001). Synergy and Other Interactions in Phytomedicines. Phytomedicine.

[37] Esatbeyoglu T., Wagner A.E., Motafakkerazad R., Nakajima Y., Matsugo S., Rimbach G. (2014). Free Radical Scavenging and Antioxidant Activity of Betanin: Electron Spin Resonance Spectroscopy Studies and Studies in Cultured Cells. Food Chem. Toxicol..

[38] Escribano J., Pedreño M.A., García-Carmona F., Muñoz R. (1998). Characterization of the Antiradical Activity of Betalains *FromBeta vulgaris* L. Roots. Phytochem. Anal..

[39] Halliwell B., Gutteridge J.M.C. (2015). Free Radicals in Biology and Medicine.

[40] Chitraju C., Walther T.C., Farese R.V. (2019). The Triglyceride Synthesis Enzymes DGAT1 and DGAT2 Have Distinct and Overlapping Functions in Adipocytes. J. Lipid Res..

[41] Liu Q., Siloto R.M.P., Lehner R., Stone S.J., Weselake R.J. (2012). Acyl-CoA:Diacylglycerol Acyltransferase: Molecular Biology, Biochemistry and Biotechnology. Prog. Lipid Res..

[42] Weiskirchen R., Weiskirchen S., Lonardo A. (2026). Lipid Droplet Dynamics in Metabolic Regulation. RSC Chem. Biol..

[43] Sztalryd C., Brasaemle D.L. (2017). The Perilipin Family of Lipid Droplet Proteins: Gatekeepers of Intracellular Lipolysis. Biochim. Biophys. Acta (BBA)—Mol. Cell Biol. Lipids.

[44] Hardie D.G., Pan D.A. (2002). Regulation of Fatty Acid Synthesis and Oxidation by the AMP-Activated Protein Kinase. Biochem. Soc. Trans..

[45] Obaseki E., Adebayo D., Bandyopadhyay S., Hariri H. (2024). Lipid Droplets and Fatty Acid-induced Lipotoxicity: In a Nutshell. FEBS Lett..

[46] Volmer R., Ron D. (2015). Lipid-Dependent Regulation of the Unfolded Protein Response. Curr. Opin. Cell Biol..

[47] Karagöz G.E., Acosta-Alvear D., Nguyen H.T., Lee C.P., Chu F., Walter P. (2017). An Unfolded Protein-Induced Conformational Switch Activates Mammalian IRE1. eLife.

[48] Kühnlenz T., Schmidt H., Uraguchi S., Clemens S. (2014). Arabidopsis Thaliana Phytochelatin Synthase 2 Is Constitutively Active in Vivo and Can Rescue the Growth Defect of the PCS1-Deficient Cad1–3 Mutant on Cd-Contaminated Soil. J. Exp. Bot..

[49] Capaci P., Barozzi F., Anglana C., Letizia F., Del Piano I., Lenucci M.S., Nisler J., Di Sansebastiano G. (2026). Pietro Integrated TIS RITA^®^ and PGRs Strategies for Enhanced in Vitro Propagation of Carob (*Ceratonia siliqua* L.). Plant Cell Tissue Organ Cult. (PCTOC).

[50] Anglana C., Barozzi F., Capaci P., Migoni D., Rojas M., Fanizzi F.P., Di Sansebastiano G.-P. (2024). Characterization of Three Species of Aquatic Mosses in Axenic Culture for Biomonitoring and Biotechnological Applications. Aquat. Bot..

[51] Castellar R., Obón J.M., Alacid M., Fernández-López J.A. (2003). Color Properties and Stability of Betacyanins from *Opuntia* Fruits. J. Agric. Food Chem..

[52] Lavanya V., Thamaraiselvi S.P., Uma D. (2019). Studies on Extraction of Betalain Pigments by Different Solvents and Assessing Antioxidant Activity of Bougainvillea Spectabilis and Celosia Argentea Flowers. Madras Agric. J..

[53] Anglana C., Rojas M., Girelli C.R., Barozzi F., Quiroz-Troncoso J., Alegría-Aravena N., Montefusco A., Durante M., Fanizzi F.P., Ramírez-Castillejo C. (2023). Methanolic Extracts of *D. Viscosa* Specifically Affect the Cytoskeleton and Exert an Antiproliferative Effect on Human Colorectal Cancer Cell Lines, According to Their Proliferation Rate. Int. J. Mol. Sci..

[54] Cataldo D.A., Maroon M., Schrader L.E., Youngs V.L. (1975). Rapid Colorimetric Determination of Nitrate in Plant Tissue by Nitration of Salicylic Acid. Commun. Soil Sci. Plant Anal..

[55] Durante M., Montefusco A., Marrese P.P., Soccio M., Pastore D., Piro G., Mita G., Lenucci M.S. (2017). Seeds of Pomegranate, Tomato and Grapes: An Underestimated Source of Natural Bioactive Molecules and Antioxidants from Agri-Food by-Products. J. Food Compos. Anal..

[56] Girelli C.R., Serio F., Accogli R., Angilè F., De Donno A., Fanizzi F.P. (2021). First Insight into Nutraceutical Properties of Local Salento Cichorium Intybus Varieties: NMR-Based Metabolomic Approach. Int. J. Environ. Res. Public Health.

[57] Carta G., Murru E., Banni S., Manca C. (2017). Palmitic Acid: Physiological Role, Metabolism and Nutritional Implications. Front. Physiol..

[58] Palomino O., Giordani V., Chowen J., Fernández-Alfonso M., Goya L. (2022). Physiological Doses of Oleic and Palmitic Acids Protect Human Endothelial Cells from Oxidative Stress. Molecules.

[59] Domínguez-López I., Arancibia-Riveros C., Casas R., Tresserra-Rimbau A., Razquin C., Martínez-González M.Á., Hu F.B., Ros E., Fitó M., Estruch R. (2022). Changes in Plasma Total Saturated Fatty Acids and Palmitic Acid Are Related to Pro-Inflammatory Molecule IL−6 Concentrations after Nutritional Intervention for One Year. Biomed. Pharmacother..

[60] Matyash V., Liebisch G., Kurzchalia T.V., Shevchenko A., Schwudke D. (2008). Lipid Extraction by Methyl-Tert-Butyl Ether for High-Throughput Lipidomics. J. Lipid Res..

[61] Giudetti A.M., Guerra F., Longo S., Beli R., Romano R., Manganelli F., Nolano M., Mangini V., Santoro L., Bucci C. (2020). An Altered Lipid Metabolism Characterizes Charcot-Marie-Tooth Type 2B Peripheral Neuropathy. Biochim. Biophys. Acta (BBA)—Mol. Cell Biol. Lipids.

