# Supplementary material for: An Aqueous Extract of Beta vulgaris subsp. Vulgaris Beetroot Group Reduces Lipid Accumulation in Human Keratinocyte Cells
Source: Int J Mol Sci. 2026 May 27;27(11):4816. doi: 10.3390/ijms27114816 (PMC13256935; doi:10.3390/ijms27114816)
Supplement: Supplementary file 1 [file ijms-27-04816-s001.zip › ijms-4279718-supplementary.pdf]

International Journal of *Molecular Sciences*

MDPI

Article

An aqueous extract of *Beta vulgaris* subsp. Vulgaris Beetroot Group reduces lipid accumulation in human keratinocyte cells

Elisa Bisconti[1], Fabrizio Barozzi[1], Erika Stefàno[1], Ilaria Serra[1,2], Francesco Vari[1], Giulia Vergine[1], Marina Damato[3], Rocco Placì[1], Francesco Paolo Fanizzi[1], Dario Domenico Lofrumento[1], Gian Pietro Di Sansebastiano[1], Francesca Baldassarre[1,4], Daniele Vergara[1], Anna Maria Giudetti[1*], Giuseppe Ciccarella[1,4,5]

[1] Department of Biological and Environmental Sciences and Technologies (DiSTeBA), University of Salento, 73100 Lecce, Italy
[2] Department of Physiology and Pharmacology "V. Erspamer", Sapienza University of Rome, P.le Aldo Moro 5, 00185, Rome, Italy
[3] Department of Experimental Medicine (Di.Me.S.), University of Salento, 73100 Lecce, Italy
[4] Institute of Nanotechnology, CNR NANOTEC, National Research Council, Via Monteroni, 73100 Lecce, Italy
[5] Department of Biological and Environmental Sciences, UdR INSTM of Lecce University of Salento, Via Monteroni, 73100 Lecce, Italy

* Correspondence: anna.giudetti@unisalento.it (A.M.G.)

Supplementary

Table S1. Chemical shifts (δ, ppm) and assignments of identified metabolites in the ^{1}H and ^{13}C NMR spectra of *B. vulgaris* Extract (BvE) (D_2O). The concentrations of metabolites found in the extract were calculated by peaks integration and TSP was used as an internal standard.

Metabolites	δ ^{1}H (ppm)	δ ^{13}C (ppm)	Concentration (mg ml^{-1})
**Alanine (Ala)**	3.78 (m)<br>1.48 (d)*	53.40<br>18.98	0.242 ± 0.024
**β-alanine (β-Ala)**	3.17 (t)<br>2.54 (t)	40.05<br>36.36	-
**Alloleucine**	1.45 (m)<br>0.98 (t)<br>0.94 (d)	24.2<br>18.25<br>13.95	-
**Arabinose**	5.27 (d)<br>4.51 (d)*	94.95<br>99.2	0.037 ± 0.002
**Arginine (Arg)**	1.91 (m)<br>1.69 (m)*	30.39<br>26.72	0.220 ± 0.055
**Asparagine (Asn)**	2.96 (dd)*<br>2.85 (dd)	37.33	0.152 ± 0.021
**Aspartate (Asp)**	2.81 (dd)<br>2.69 (dd)	39.30	-
**Betaine**	3.90 (s)<br>3.27 (s)*	69.08<br>56.25	0.085 ± 0.009
**Betanin**	7.15 (s)*<br>7.07 (s)	102.51<br>116.77	0.307 ± 0.037
**Choline (Cho)**	4.06 (m)<br>3.52 (m)<br>3.20 (s)*	58.42<br>70.30<br>56.77	0.032 ± 0.001
**Formate**	8.46 (s)*	170.72	0.001 ± 0.001
**Fructose (Fru)**	4.12 (d)*<br>4.03 (d)<br>4.00 (m)<br>3.57 (t)	78.56<br>66.25<br>72.09<br>66.77	1.045 ± 0.318
**Fucose**	1.24 (d)<br>1.19 (d)*	17.11	0.013 ± 0.001

**Fumarate**	6.52 (s)*	139.12	0.002 ± 0.001
**Galactose**	5.27 (d)*<br>4.58 (d)<br>3.98 (d)<br>3.94 (d)	95.39<br><br>74.32<br>73.33	0.033 ± 0.015
**Gluconate (Gluc)**	4.13 (d)*<br>4.06 (t)	79.03<br>75.05	0.094 ± 0.050
**α-Glucose**	5.24 (d)*<br>3.22-3.90	95.00	3.795 ± 2.914
**β-Glucose**	4.65 (d)*<br>3.22-3.90	94.30	3.409 ± 2.525
**Glutamate (Glu)**	2.36 (m)*<br>2.06 (m)	36.29<br>29.66	0.140 ± 0.074
**Glutamine (Glu)**	3.78 (m)<br>2.46 (m)*<br>2.14 (m)	57.02<br>33.69<br>29.03	5.367 ± 0.253
**Isoleucine (Ile)**	1.42 (m)<br>1.01 (d)<br>0.95 (t)	-<br>19.49<br>27.17	0.009 ± 0.001
**Leucine (Leu)**	1.70 (m)<br>0.96 (m)	26.74<br>24.87	-
**O-Acetylcarnitine (ALCAR)**	3.64 (m)<br>3.55 (m)<br>3.18 (s)	 70.46<br>56.92	-
**Phenylalanine (Phe)**	7.38 (m)*	127.90<br>133.60	0.004 ± 0.002
**Propionate**	2.17 (m)<br>1.02 (t)	34.07<br>17.55	-
**Pyroglutamate (p-Glu)**	4.18 (dd)*<br>2.52 (m)<br>2.39 (m)<br>2.02 (m)	61.22<br>28.33<br>32.60<br>26.61	0.107 ± 0.027
**Riboflavin**	7.99 (s)*<br>7.97 (s)	131.64<br>120.36	0.020 ± 0.009

**Sucrose**	5.37 (d)*	94.98	0.004 ± 0.001
**Serine (Ser)**	3.97 (m)*	92.91	-
**Threonine (Thr)**	4.26 (m)<br>1.33 (d)*	68.85<br>22.29	0.028 ± 0.001
**Tryptophan (Trp)**	7.74 (d)*<br>7.54 (d)<br>7.32 (s)<br>7.29 (t)<br>7.20 (t)	121.34<br>114.80<br>127.93<br>125.05<br>122.39	0.046 ± 0.009
**Trigonelline**	9.12 (s)*<br>8.84 (m)<br>8.08 (t)<br>4.44 (s)	140.98<br>147.68<br>131.79<br>51.55	0.005 ± 0.014
**Tyrosine (Tyr)**	7.19 (d)*<br>6.90 (d)	133.63<br>118.70	0.036 ± 0.012
**Uridine**	7.87 (d)*<br>5.91 (m)	144.79<br>105.22	0.020 ± 0.005
**Valine (Val)**	2.27 (m)<br>1.05 (d)<br>0.99 (d)*	31.87<br>20.81<br>19.46	0.033 ± 0.005
**2-Hydroxyisobutyrate (2-HB)**	1.36 (s)*	29.35	0.005 ± 0.003
**3-Hydroxybutyrate (3-HB)**	1.22 (d)	24.21	-
**4-Aminobutyrate (GABA)**	3.02 (t)*<br>2.30 (t)<br>1.90 (m)	42.16<br>37.19<br>26.42	0.082 ± 0.015

Table S2. List of primary antibodies used for Western Blot analyses.

Antibody	Source	Brand	Cat.no
**ACC**	Rabbit	Cell signaling	#3662S
**ATF6**	Rabbit	Cell Signaling	#65880s
**ATGL**	Rabbit	Cell Signaling	#2138s
**Catalase**	Mouse	Santa Cruz	sc-271803
**CPT1A**	Rabbit	Cell Signaling	#12252S
**DGAT1**	Rabbit	Santa Cruz	sc-32861
**FASN**	Rabbit	Cell Signaling	#3180S
**GPx-4**	Mouse	Santa Cruz	sc-166570
**GRP78**	Rabbit	Cell Signaling	#3177s
**MGAL**	Rabbit	GeneTex	GTXGT637775
**NRF2**	Rabbit	Antibodies	A308758
**P53**	Rabbit	Cell Signaling	#30313s
**P62**	Rabbit	Cell Signaling	5114S
**pACC**	Rabbit	Cell Signaling	#3661S
**pAMPK substrate**	Rabbit	Cell Signaling	#5759S
**PERK**	Mouse	Santa Cruz	sc-377400
**PLIN2**	Rabbit	Cell Signaling	#45535s
**pP53**	Rabbit	Cell Signaling	#82530s
**PPARα**	Mouse	Santa Cruz	sc-398394
**SOD-1**	Mouse	Santa Cruz	sc-17767
**SREBP-1**	Mouse	Santa Cruz	sc-365513
**Total OXPHOS**	Mouse	ABCAM	ab110413
**XBP1-s**	Rabbit	Cell Signaling	#40435S

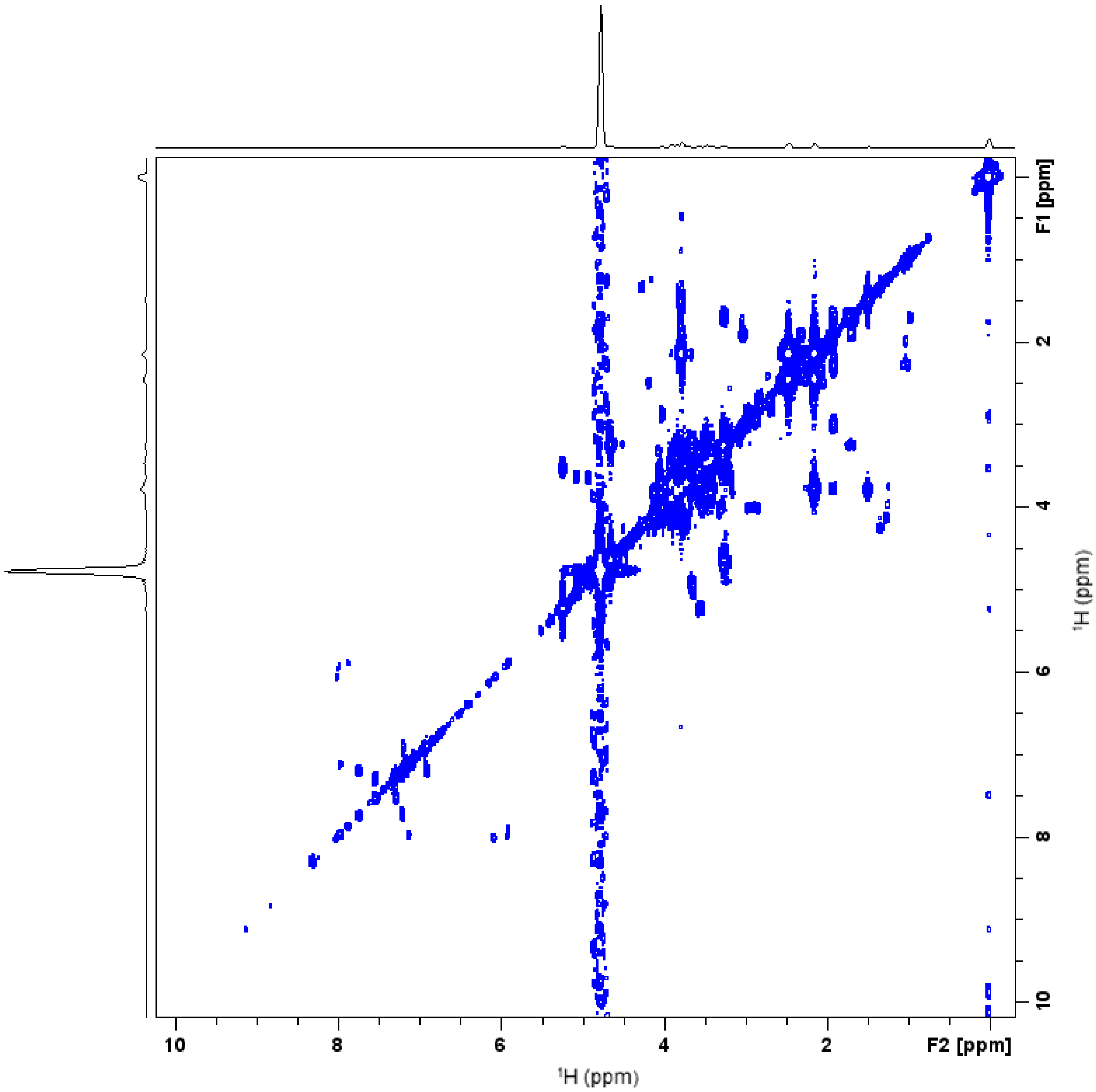

Figure S1. Two-dimensional ¹H, ¹H-COSY NMR spectrum of *B. vulgaris* Extract (BvE) in D_2O.

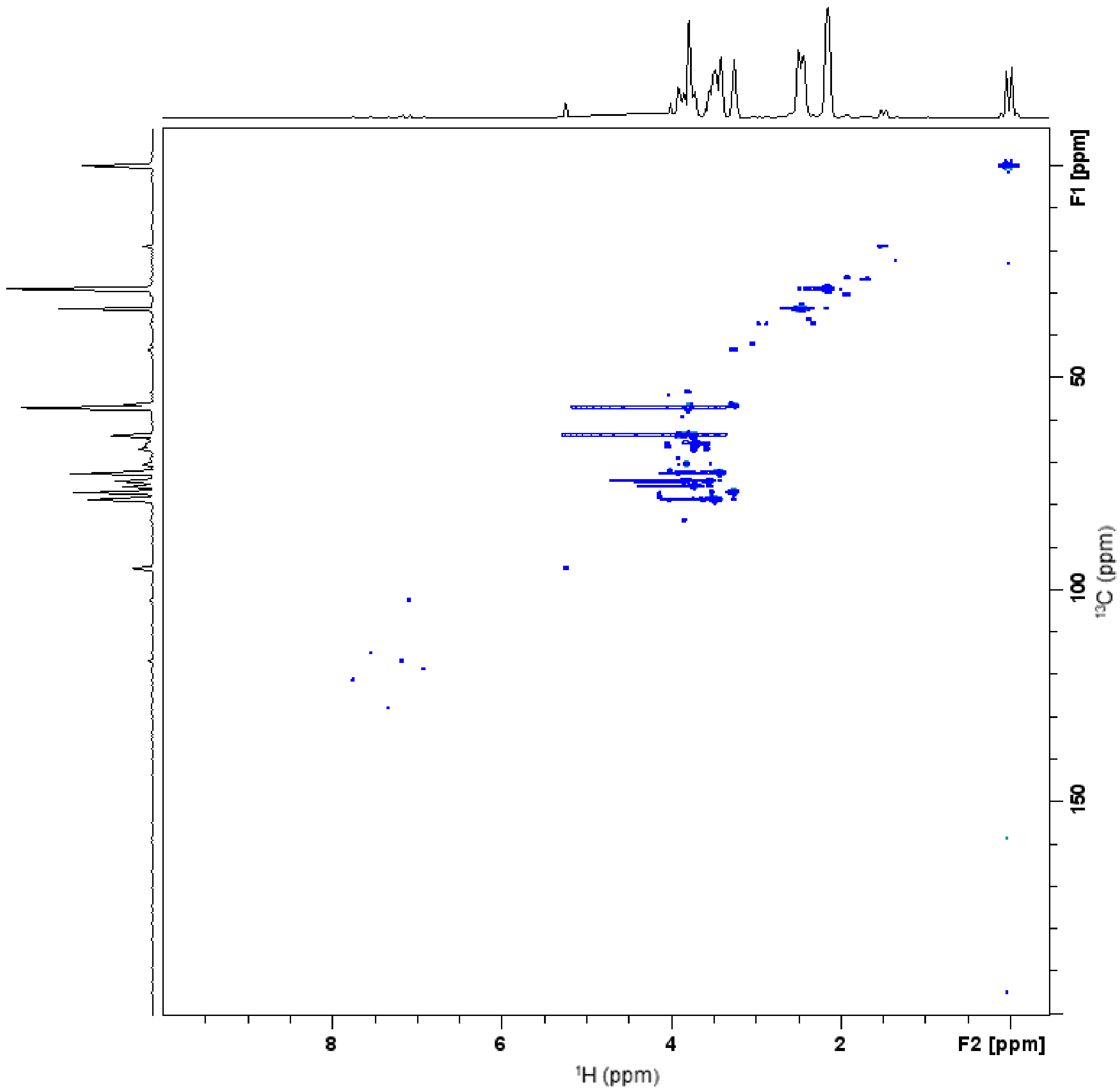

Figure S2. Two-dimensional ^1H, ^{13}C-HSQC spectrum of *B. vulgaris* Extract (BvE) in D_2O.

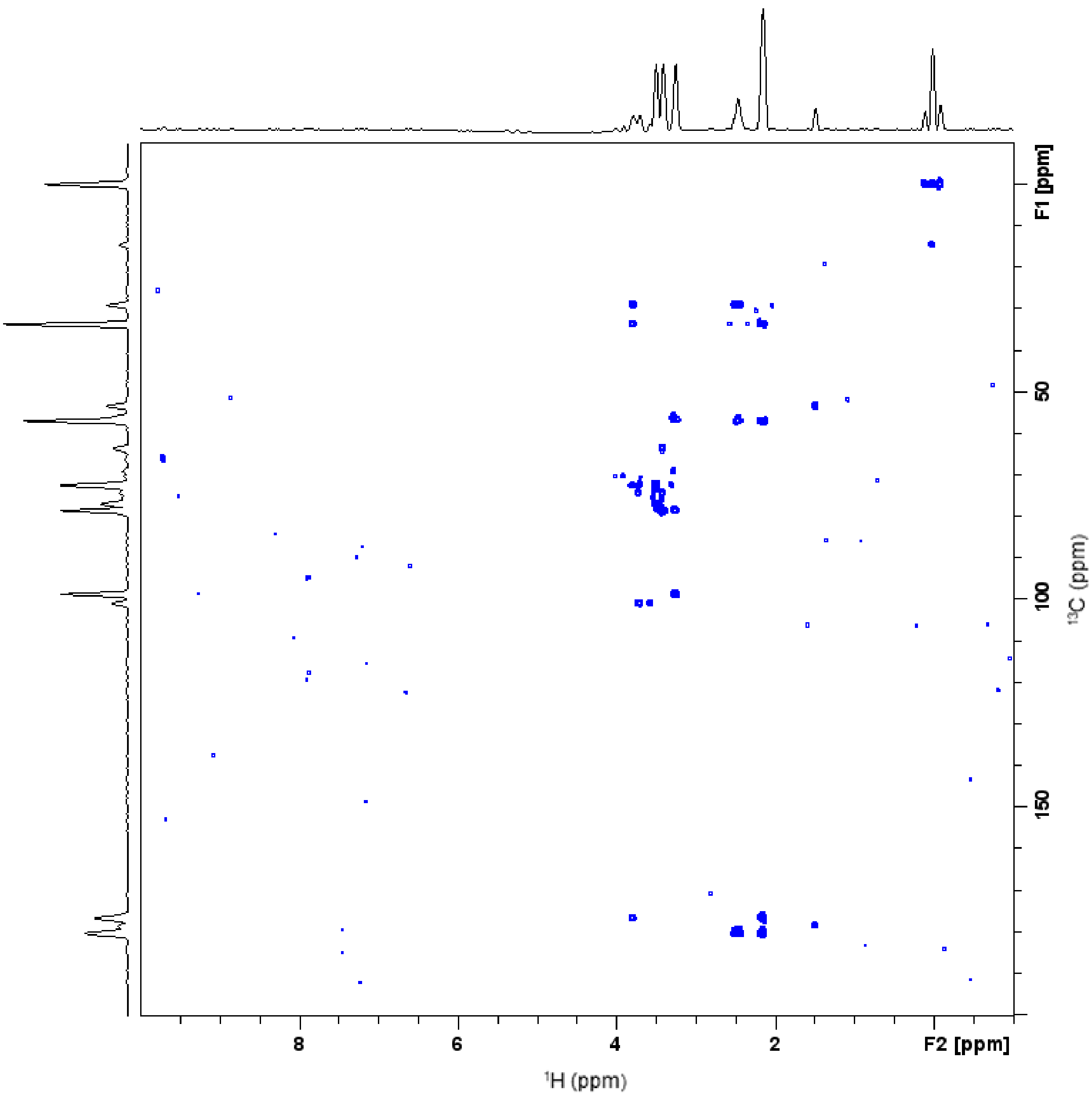

Figure S3. Two-dimensional ^{1}H, ^{13}C-HMBC spectrum of *B. vulgaris* Extract (BvE) in D_2O.

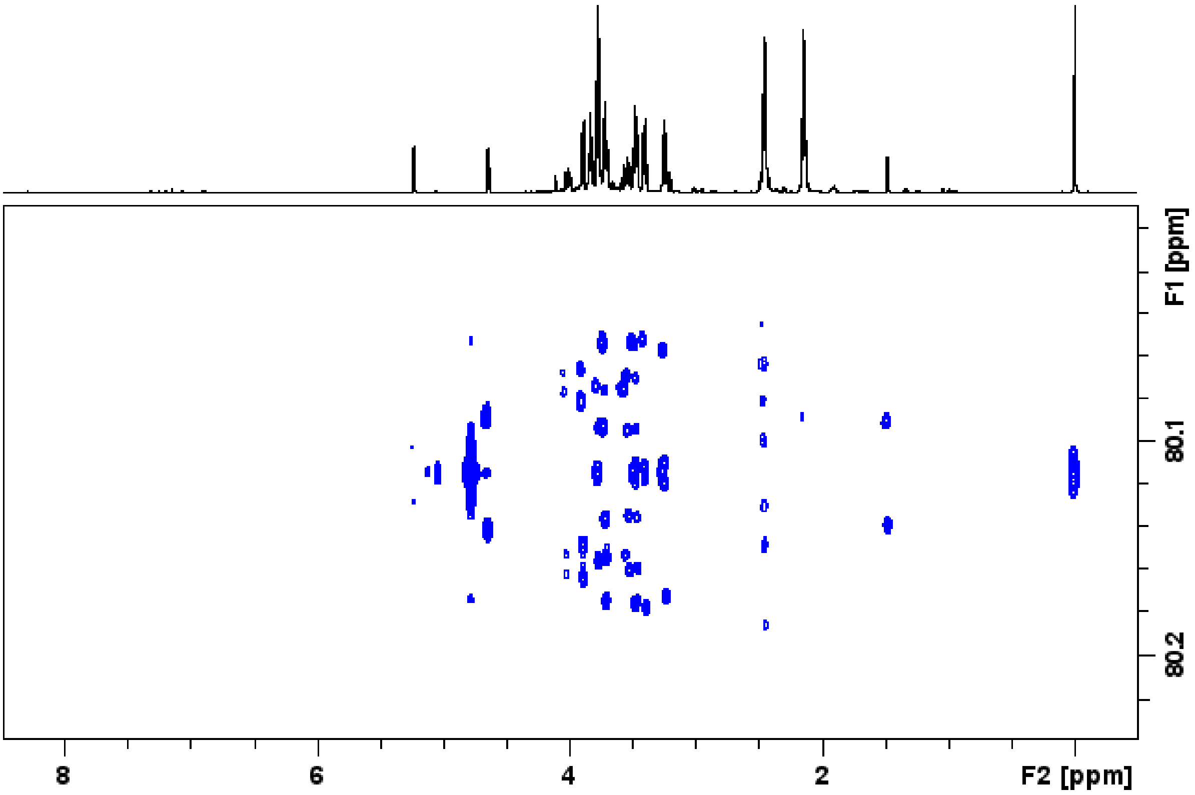

Figure S4. Two-dimensional ^{1}H, ^{1}H J-resolved spectrum of B. vulgaris Extract (BvE) in D_2O.

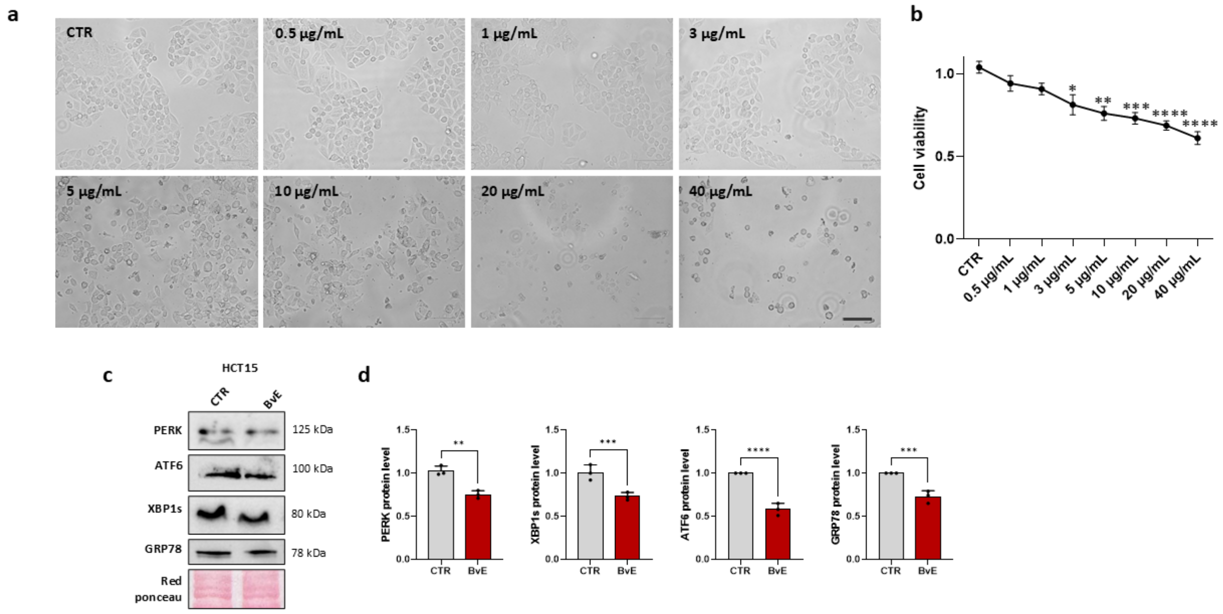

Figure S5. Effect of BvE on cell viability and ER stress in HCT15 cells. **(a)** Representative bright-field microscopy images of HCT15 cells, without treatment (CTR, control) and after 24 h of treatment with different concentrations of *Beta vulgaris* extract (BvE) (0.5, 1, 3, 5, 10, 20, and 40 µg/mL). **(b)** Cell viability assay performed on HCT15 cells treated for 24 h with BvE at concentrations of 0.5, 1, 3, 5, 10, 20, and 40 µg/mL. **(c)** Western blot analysis of PERK, ATF6, XBP1s, and GRP78 protein levels and **(d)** relative quantification in HCT15 cells treated with BvE (1 µg/mL). Ponceau S staining was used as a total protein loading control. Scale bars 100 µm. Data are shown as mean ± SD of at least three independent experiments (*p<0.05; **p<0.01; ***p<0.005; ****p<0.001).

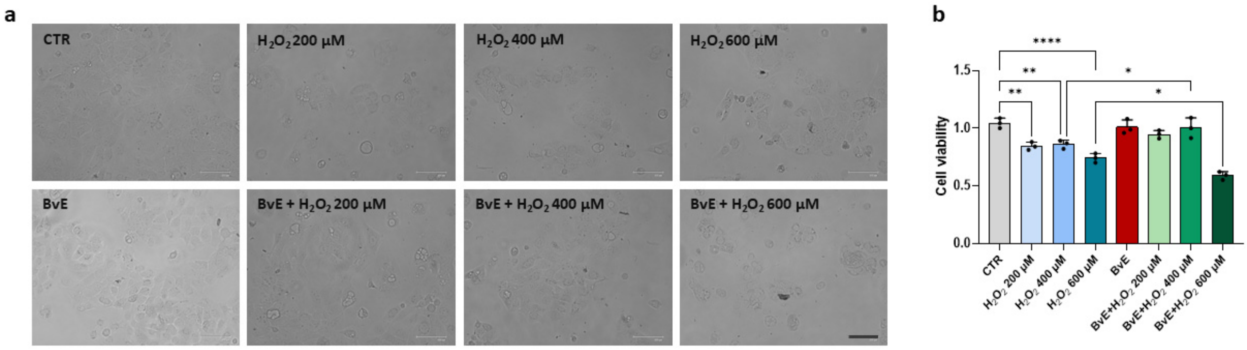

Figure S6. BvE attenuates H_2O_2-induced loss of viability in HaCaT cells. **(a)** Representative bright-field microscopy images and **(b)** cell viability assay of HaCaT cells exposed to increasing doses of hydrogen peroxide (H_2O_2; 200, 400, and 600 μM) for 24 h, in the presence or absence of *Beta vulgaris* extract (BvE; 1 μg/mL). Scale bars 100 μm. Data are shown as mean ± SD of at least three independent experiments (*p<0.05; **p<0.01; ***p<0.005; ****p<0.001).

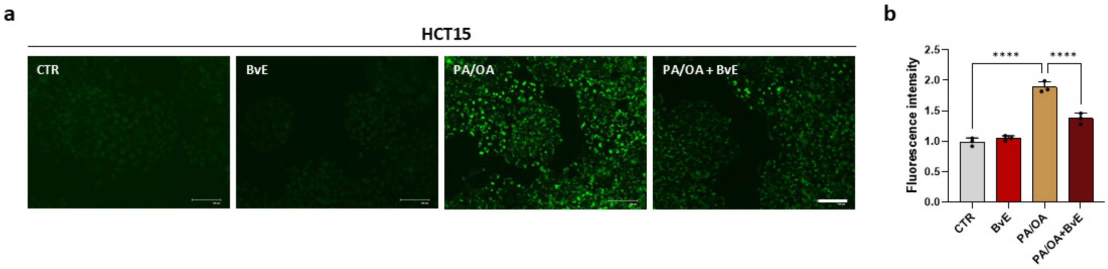

Figure S7. BvE reduces lipid droplet accumulation in HCT15 cells. **(a)** Representative fluorescence images showing lipid droplets (LDs, green) stained with BODIPY probe and **(b)** relative fluorescence intensity in HCT15 cell line treated with *Beta vulgaris* extract (BvE; 1 µg/mL) for 24 h, palmitic acid (PA) and oleic acid (OA) for 48 h (PA/OA; 1:1 ratio, 200 µM each), and pre-treatment of PA/OA for 48 h + BvE for 24 h (co-treatment PA/OA+BvE). Scale bars 100 µm. Data are shown as mean ± SD of at least three independent experiments (*p<0.05; **p<0.01; ***p<0.005; ****p<0.001).
